# TNF Induces Pathogenic Programmed Macrophage Necrosis in Tuberculosis through a Mitochondrial-Lysosomal-Endoplasmic Reticulum Circuit

**DOI:** 10.1016/j.cell.2019.08.004

**Published:** 2019-09-05

**Authors:** Francisco J. Roca, Laura J. Whitworth, Sarah Redmond, Ana A. Jones, Lalita Ramakrishnan

**Affiliations:** 1 Molecular Immunity Unit, Department of Medicine, University of Cambridge, MRC Laboratory of Molecular Biology, Cambridge CB2 OQH, UK; 2 Department of Microbiology, University of Washington, Seattle, WA 98195, USA

**Keywords:** TNF-mediated necrosis, macrophage, tuberculosis, mitochondrion, ROS, Calcium, BAX, ER, ryanodine receptors, calcium channel blockers

## Abstract

Necrosis of infected macrophages constitutes a critical pathogenetic event in tuberculosis by releasing mycobacteria into the growth-permissive extracellular environment. In zebrafish infected with *Mycobacterium marinum* or *Mycobacterium tuberculosis*, excess tumor necrosis factor triggers programmed necrosis of infected macrophages through the production of mitochondrial reactive oxygen species (ROS) and the participation of cyclophilin D, a component of the mitochondrial permeability transition pore. Here, we show that this necrosis pathway is not mitochondrion-intrinsic but results from an inter-organellar circuit initiating and culminating in the mitochondrion. Mitochondrial ROS induce production of lysosomal ceramide that ultimately activates the cytosolic protein BAX. BAX promotes calcium flow from the endoplasmic reticulum into the mitochondrion through ryanodine receptors, and the resultant mitochondrial calcium overload triggers cyclophilin-D-mediated necrosis. We identify ryanodine receptors and plasma membrane L-type calcium channels as druggable targets to intercept mitochondrial calcium overload and necrosis of mycobacterium-infected zebrafish and human macrophages.

## Highlights

• TNF induces mitochondrial ROS to cause necrosis of mycobacterium-infected macrophages
• Mitochondrial ROS activate lysosomal enzymes that lead to BAX activation
• BAX activates ER ryanodine receptors to cause Ca^2+^ flow into the mitochondrion
• Drugs preventing mitochondrial Ca^2+^ overload prevent pathogenic macrophage necrosis in TB

## Graphical Abstract

**Figure:**
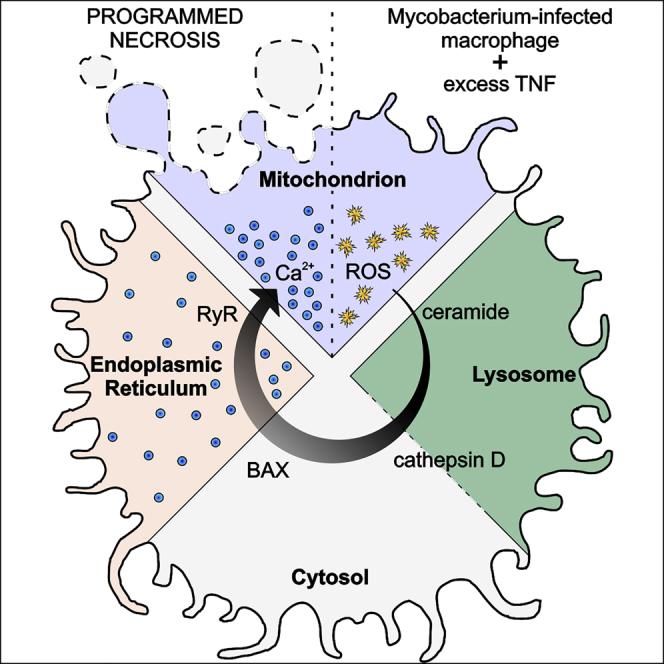

## Teaser

During TB infection, the necrosis of infected macrophages involves an inter organelle signaling relay involving druggable targets such as ryanodine receptors and plasma membrane L-type Ca^2+^ channels.

## Introduction

The pathogenic life cycle of *Mycobacterium tuberculosis* (Mtb), the agent of human tuberculosis (TB), is fueled by its multiple interactions with host macrophages (Cambier et al., 2014, Srivastava et al., 2014). Mycobacteria use macrophages to traverse host epithelial barriers to enter deeper tissues, where they recruit additional macrophages to form granulomas, pathognomonic structures that can serve as intracellular bacterial growth niches (Cambier et al., 2014, Ramakrishnan, 2012). Granuloma macrophages can undergo necrosis, a key pathogenic event that further increases bacterial growth in the more permissive extracellular milieu (Divangahi et al., 2013, Ramakrishnan, 2012), thereby increasing disease morbidity and transmission (Cambier et al., 2014, Huang et al., 2014, Reichler et al., 2002).

Mycobacterium-macrophage interactions and resultant macrophage fates can be detailed in the optically transparent zebrafish larva infected with *Mycobacterium marinum* (Mm), a close genetic relative of Mtb (Pagán and Ramakrishnan, 2014, Takaki et al., 2013). In this model, distinct host genetic mutations that increase macrophage necrosis render the host hypersusceptible by promoting unrestricted extracellular mycobacterial growth (Berg et al., 2016, Clay et al., 2008, Pagán et al., 2015, Tobin et al., 2012). One genetic perturbation that produces hypersusceptibility through macrophage necrosis increases expression of leukotriene A4 hydrolase (LTA4H), which catalyzes the final step in the synthesis of the inflammatory lipid mediator leukotriene B4 (LTB_4_) (Tobin et al., 2012). Humans with a functional *LTA4H* promoter variant that increases LTA4H expression are also hypersusceptible to TB (Thuong et al., 2017, Tobin et al., 2012). Among cases of tuberculous meningitis, the severest form of TB, *LTA4H*-high individuals had increased risk of death. Consistent with inflammation-induced mortality, survival was dramatically increased among patients who received adjunctive anti-inflammatory therapy with glucocorticoids (Thuong et al., 2017, Tobin et al., 2012).

**Figure 1:**
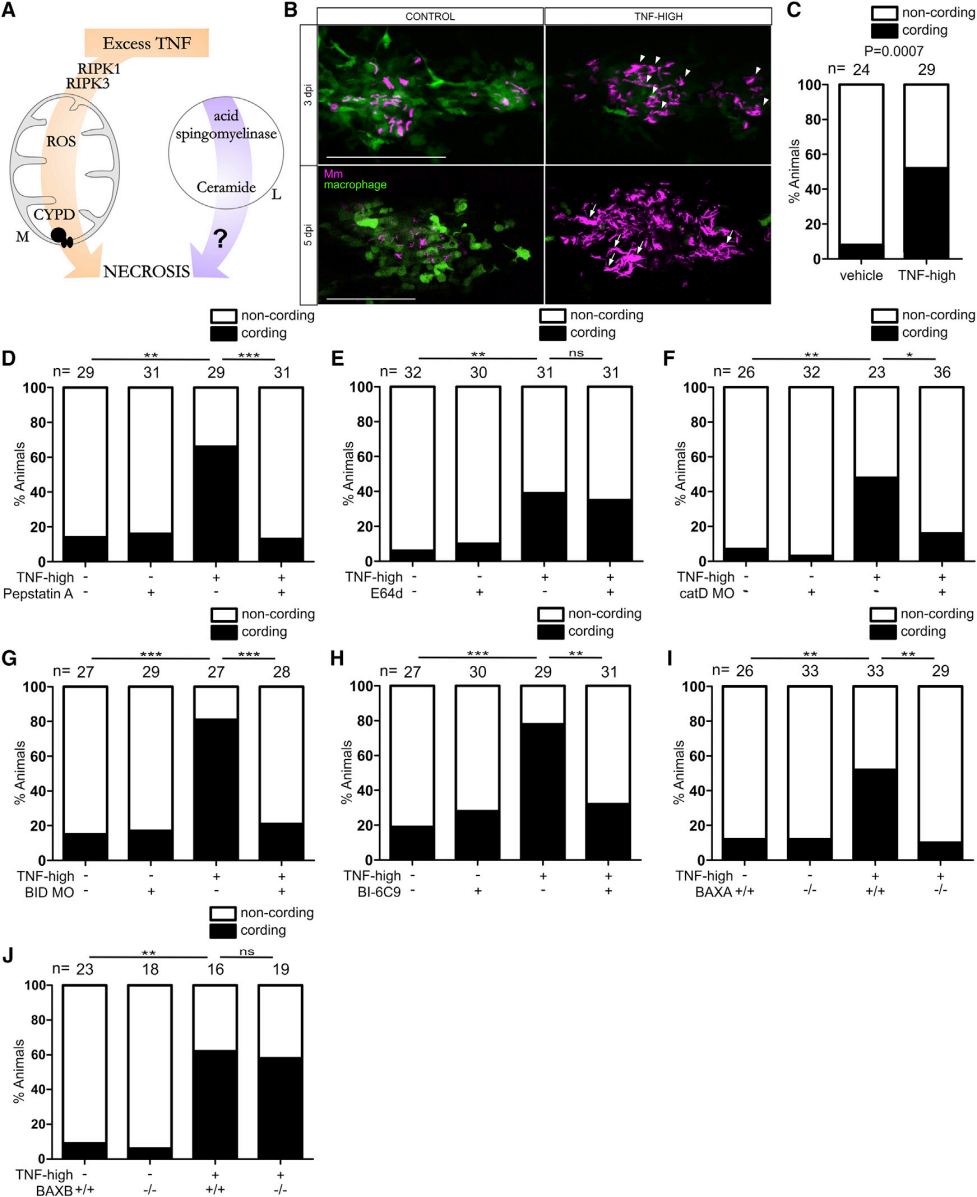
Ceramide Causes Necrosis through Cathepsin D, BID, and BAX (A) Cartoon of TNF-mediated necrosis pathway components. CYPD, cyclophilin D; M, mitochondrion; L, lysosome. (B) Confocal images of granulomas in 3 or 5 dpi TNF-high or control larvae with yellow fluorescent macrophages infected with red fluorescent Mm. Arrowheads, extracellular bacteria; arrows, extracellular, cording bacteria. Scale bar, 100 μm. (C) Cording in 5 dpi TNF-high and control larvae. (D) Cording in 5 dpi TNF-high or control larvae treated with pepstatin A. (E) Cording in 5 dpi TNF-high or control larvae treated with E64d. (F) Cording in 5 dpi TNF-high and control larvae that are wild-type (WT) or cathepsin D morphant. (G) Cording in 5 dpi TNF-high and control larvae that are WT or BID morphant. (H) Cording in 5dpi TNF-high and control larvae treated with BI-6C9. (I) Cording in 5 dpi TNF-high or control larvae that are WT or BAXA mutant. (J) Cording in 5 dpi TNF-high and control larvae that are WT or BAXB mutant. (C–J) ^∗^p < 0.05; ^∗∗^p < 0.01; ^∗∗∗^p < 0.001 (Fisher’s exact test). Each panel representative of 3–6 independent experiments. See also Figure S1.

The human relevance of the zebrafish findings provided the impetus to carry out a detailed mechanistic dissection of the necrosis pathway. In the zebrafish, we showed that susceptibility of the LTA4H-high state is due to the excessive production of the pro-inflammatory cytokine tumor necrosis factor (TNF) that, at optimal levels, is host protective (Tobin et al., 2012). Excess TNF triggers RIPK1- and RIPK3-dependent programmed necrosis of mycobacterium-infected macrophages, but not uninfected macrophages in the same animal (Roca and Ramakrishnan, 2013). TNF-RIPK1-RIPK3 interactions increase mitochondrial reactive oxygen species (ROS) production, which are required for necrosis along with cyclophilin D, a mitochondrial matrix protein (Roca and Ramakrishnan, 2013). Oxidative stress can activate cyclophilin D, which promotes sustained opening of the mitochondrial permeability transition pore complex (mPTP); this leads to disruption of the membrane potential and ATP depletion (Baines, 2010, Bernardi, 2013, Halestrap et al., 2004, Nakagawa et al., 2005). The dual requirement for mitochondrial ROS and cyclophilin D would be consistent with a mitochondrion-intrinsic necrosis pathway where TNF-induced mitochondrial ROS activate cyclophilin D (Figure 1A).

However, we found that necrosis also requires lysosomal components, specifically ceramide produced by lysosomal acid sphingomyelinase (aSM) (Roca and Ramakrishnan, 2013) (Figure 1A). In this work, we have sought to understand why lysosomal ceramide would be required for mitochondrially mediated necrosis (Figure 1A). We show that TNF-mediated necrosis results from a single pathway, an inter-organellar circuit that initiates in the mitochondrion and traverses the lysosome, cytosol, and endoplasmic reticulum (ER) before returning to the mitochondrion to execute necrosis. This intricate circuit that begins with mitochondrial ROS production ultimately results in mitochondrial Ca^2+^ overload, which is a likely trigger for cyclophilin D activation (Baines et al., 2005, Nakagawa et al., 2005). We find that Mtb, like Mm, triggers this pathway in the zebrafish, and both Mm and Mtb cause TNF-mediated necrosis of human macrophages. Elucidating this pathway has led to the identification of multiple commonly used drugs that inhibit TNF-induced necrosis of mycobacterium-infected macrophages by preventing mitochondrial Ca^2+^ overload.

## Results

### TNF-Mediated Necrosis Is Dependent on Cathepsin D, BID, and BAX

Why might necrosis, which could be completed in a mitochondrion-intrinsic fashion (Baines, 2010, Bernardi, 2013, Zamzami et al., 2005), require lysosomal components? The literature revealed a series of clues to this conundrum. Lysosomal ceramide mediates TNF-induced caspase-independent programmed cell death in a fibroblast cell line (Dumitru and Gulbins, 2006, Heinrich et al., 2004, Taniguchi et al., 2015, Thon et al., 2005). *In vitro*, ceramide produced by acid sphingomyelinase activates lysosomal proteases, including cathepsins B and D (Heinrich et al., 1999, Heinrich et al., 2004, Taniguchi et al., 2015). In cell-free assays and in cultured cells, cathepsins B and D cleave the pro-apoptotic protein BID to its active form tBID (Appelqvist et al., 2012, Cirman et al., 2004, Droga-Mazovec et al., 2008, Heinrich et al., 2004). tBID activates BAX, an effector of apoptosis (Westphal et al., 2014), and the cancer chemotherapy drug gemcitabine promotes BAX-dependent apoptosis of a glioma cell line via lysosomal ceramide accumulation and cathepsin D activation (Dumitru et al., 2009). BAX is reported to work in conjunction with cyclophilin D to induce mitochondrion-intrinsic necrosis (Karch et al., 2013, Karch et al., 2017, Whelan et al., 2012). Collectively, these findings led us to consider that lysosomal components mediate necrosis; lysosomal ceramide activates cathepsin B and/or cathepsin D, which in turn activate(s) BID and thereby recruits BAX.

If this sequence is operant, then inhibiting cathepsin B and/or D, BID, and BAX should inhibit TNF-mediated macrophage necrosis. To assess this, we injected larvae with TNF 1-day post infection (dpi) to create TNF-high animals (see STAR Methods). TNF-high animals display loss of granuloma cellularity at 2–3 days that renders bacteria extracellular (Figure 1B, compare control granuloma in top left panel to TNF-high granuloma in top right panel). Extracellular bacteria grow more profusely (Figure 1B, compare growth from 3 to 5 dpi in control and TNF-high granulomas), with a characteristic cording morphology (Figure 1B, bottom right panel). The presence of bacterial cording is a reliable, facile, quantifiable indicator that granuloma macrophage necrosis has occurred within the animal (Clay et al., 2008, Tobin et al., 2012)(Figure 1C). We inhibited cathepsins B and D, respectively, using the cysteine cathepsin inhibitor E64d and the aspartyl cathepsin inhibitor pepstatin A (Baldwin et al., 1993, Berg et al., 2016). Only pepstatin A inhibited TNF-mediated necrosis, pinpointing cathepsin D (Figures 1D and 1E). We confirmed cathepsin D’s involvement with genetic cathepsin D depletion using an antisense morpholino (Follo et al., 2011): cathepsin D morphants were resistant to TNF necrosis (Figure 1F). Morpholino-mediated BID knockdown and pharmacological inhibition with BI-6C9, a small molecule tBID inhibitor (Becattini et al., 2004) also inhibited necrosis (Figures 1G and 1H).

**Figure S1:**
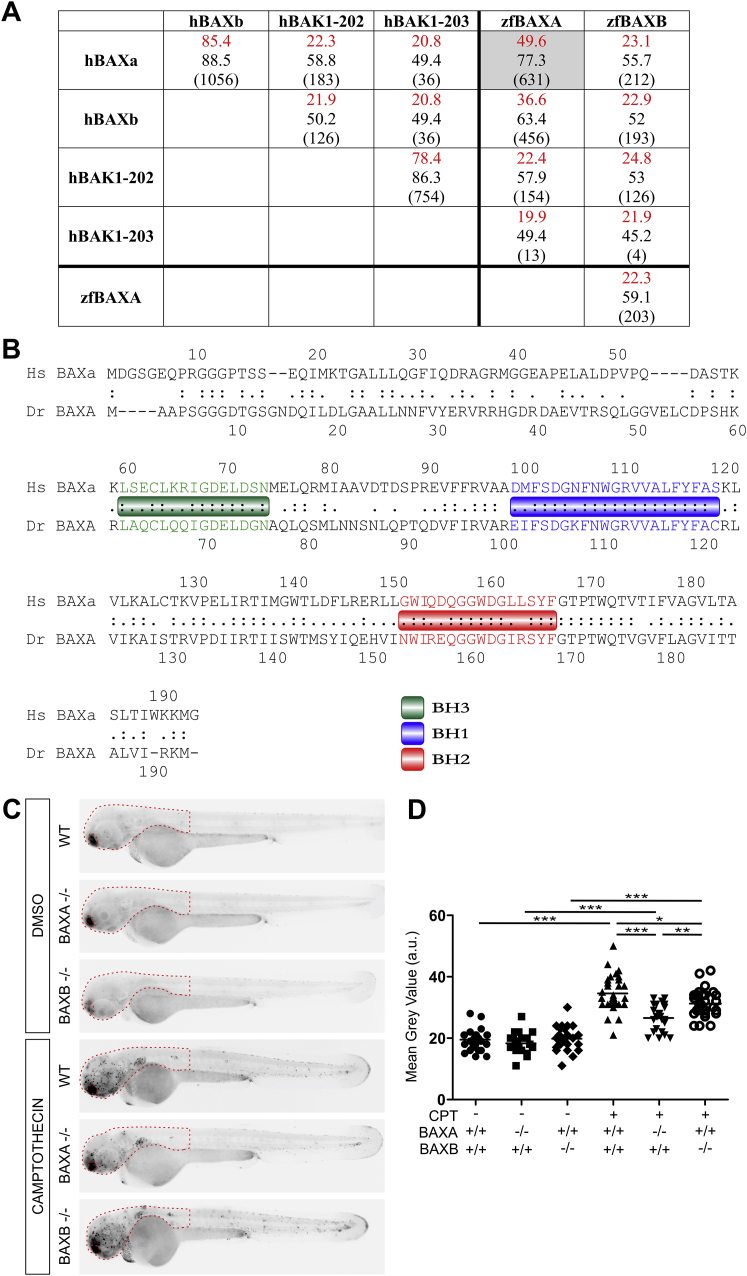
Camptothecin-Induced Apoptosis Is Decreased in BAXA and BAXB Mutant Zebrafish, Related to Figure 1 (A) Global analysis without end-gap penalty of protein sequence homology between human BAK1 (transcripts 202 and 203), BAX (only major transcripts, alpha and beta) and zebrafish BAXA and BAXB. Prefix h, human; prefix zf, zebrafish. Although BAXB has been suggested to be the zebrafish functional equivalent of human BAK (Kratz et al., 2006), neither it nor BAXA had significant homology to either human BAK transcript. Identity (red) (%), similarity (%), (global/local score). (B) Comparison of protein sequence homology between human BAX alpha and zebrafish BAXA. Relevant BH domains of BAX are showed in colored boxes. (C) Representative inverted fluorescence images of 2 dpf larvae that are WT, BAXA mutant or BAXB mutant treated with camptothecin and incubated with acridine orange to detect apoptotic cells. (D) Quantification of camptothecin-induced apoptosis (See STAR Methods) in WT, BAXA mutant or BAXB mutant fish from (C) in the area within red dashed line. ^∗^p < 0.05; ^∗∗^p < 0.01; ^∗∗∗^p < 0.001 (one-way ANOVA with Tukey’s post-test). Representative of 3 independent experiments.

Next, we asked if BAX was involved. There are two zebrafish paralogs of human BAX, BAXA and BAXB, with BAXA more similar to human BAX (Kratz et al., 2006) (Figures S1A and S1B). We tested the roles of the two BAX homologs in camptothecin-induced apoptosis, a BAX-dependent process in mammals (Albihn et al., 2007). Zebrafish BAXA mutants displayed the greater reduction in apoptosis (Figures S1C and S1D). Furthermore, BAXA mutants were resistant to TNF-mediated necrosis whereas BAXB mutants were susceptible (Figures 1I and 1J). These results show that BAX, specifically BAXA, is required for necrosis. We will refer to BAXA as BAX hereon. In sum, our findings are consistent with lysosomal ceramide mediating necrosis through cathepsin D activation leading to BID and thereby BAX activation.

### BAX’s Role in TNF-Mediated Necrosis Is Independent of Oligomerization

**Figure 2:**
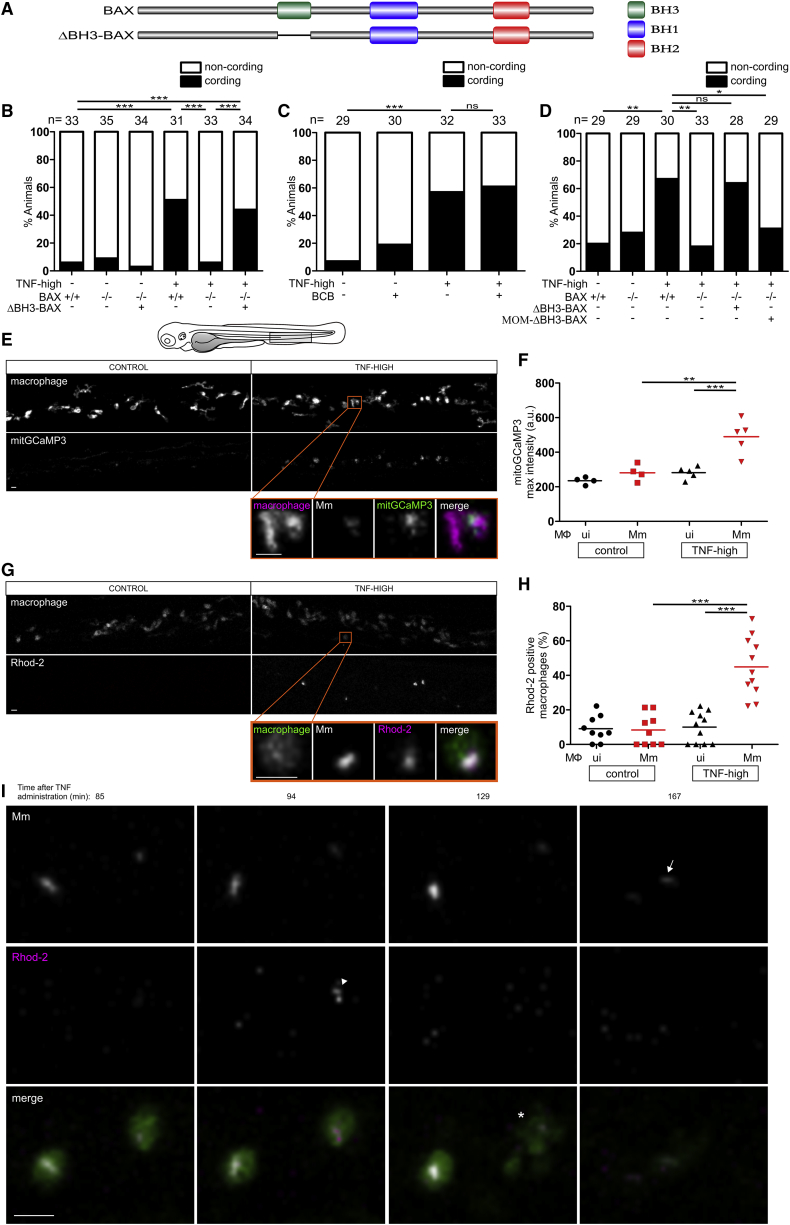
BAX Mediates Macrophage Necrosis by Promoting Mitochondrial Ca^2+^ Overload Independent of BH3-Dependent Oligomerization and Interaction with the Mitochondrial Outer Membrane (A) Schematic of BAX to show BH domains. (B) Cording in 5 dpi TNF-high and control larvae that are WT, BAX mutant, or BAX mutant expressing ΔBH3-BAX. ^∗∗∗^p < 0.001 (Fisher’s exact test). Representative of 6 independent experiments. (C) Cording in 5 dpi TNF-high and control larvae treated with BAX channel blocker (BCB). ^∗∗∗^p < 0.001 (Fisher’s exact test). Representative of 3 independent experiments. (D) Cording in 5 dpi TNF-high and control larvae that are WT, BAX mutant, BAX mutant expressing ΔBH3-BAX, or BAX mutant expressing ΔBH3-BAX targeted to the mitochondrial outer membrane (MOM-ΔBH3-BAX). ^∗^p < 0.05; ^∗∗^p < 0.01 (Fisher’s exact test). Representative of 2 independent experiments. (E) Representative confocal images of 1 dpi TNF-high (90 min post-TNF treatment) or control larvae, both expressing mitGCaMP3 (GCaMP3 targeted to the mitochondria) in their macrophages, which are red fluorescent. Area shown corresponds to area in the shaded rectangle in the cartoon above. Detailed image of orange rectangle in TNF-high larva additionally shows the bacteria within a mitGCaMP3-expressing macrophage. Scale bars, 10 μm. (F) Quantitation of mitGCaMP3 fluorescence in individual macrophages from larvae in (A). Black and red symbols represent uninfected (ui) and Mm-infected macrophages, respectively, in the same control or TNF-treated animal. Horizontal bars, means; ^∗∗∗^p < 0.001 (one-way ANOVA with Tukey’s post-test). Representative of 2 independent experiments. (G) Representative confocal images of 1 dpi TNF-high (120 min post-TNF treatment) or control larvae with yellow fluorescent macrophages showing Rhod-2 fluorescence (red). Area shown similar to (A). Detail: macrophage in the orange rectangle. Scale bar, 10 μm. (H) Percentage of uninfected or Mm-infected macrophages with Rhod-2 fluorescence from TNF-high larvae in (C). Black and red symbols represent uninfected and Mm-infected macrophages, respectively, in the same control or TNF-treated animal. Horizontal bars, means; ^∗∗∗^p < 0.001 (one-way ANOVA with Tukey’s post-test). Representative of 2 independent experiments. (I) Time-lapse confocal images of two infected macrophages in a 1 dpi larva at indicated time points after TNF administration. Arrowhead, Rhod-2 positive macrophage; asterisk, dead macrophage; arrow, extracellular bacteria. Scale bar, 10 μm. See also Figure S2.

**Figure S2:**
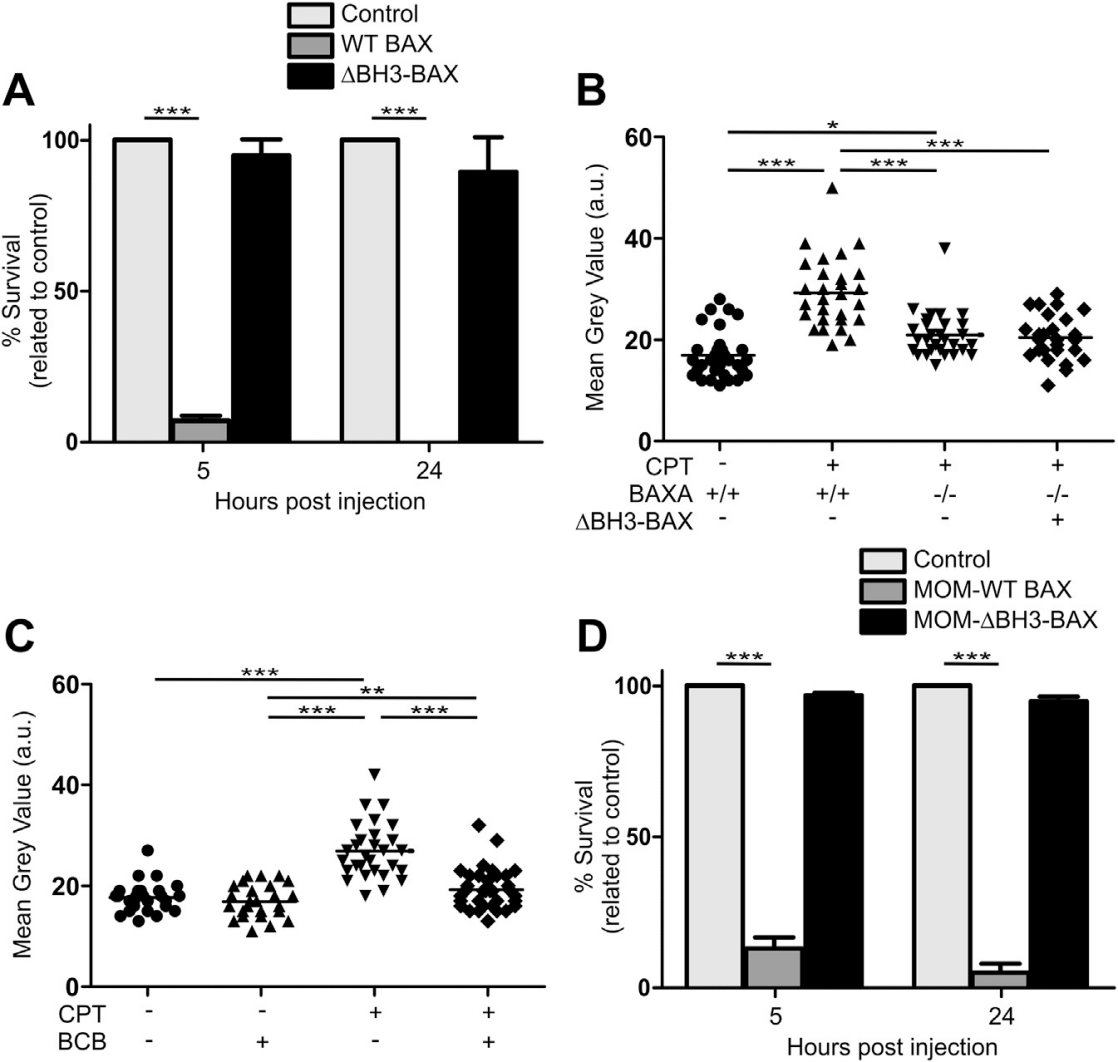
BH3 Domain of BAX Is Required for Apoptosis, Related to Figure 2 (A) Percentage survival of 5 and 24 hpf BAX mutant larvae expressing WT BAX or ΔBH3-BAX. ^∗∗∗^p < 0.001 (one-way ANOVA with Tukey’s post-test). Mean of 3 experiments (±SEM) is represented. (B) Quantification of camptothecin-induced apoptosis in 2 dpf larvae that are WT, BAXA mutant or BAXA mutant expressing ΔBH3-BAX. Horizontal bars, means; ^∗^p < 0.05; ^∗∗∗^p < 0.001 (one-way ANOVA with Tukey’s post-test). Representative of 2 independent experiments. (C) Quantification of camptothecin-induced apoptosis in 2 dpf larvae treated with BCB. Horizontal bars, means; ^∗∗^p < 0.01; ^∗∗∗^p < 0.001 (one-way ANOVA with Tukey’s post-test). Representative of 2 independent experiments. (D) Percentage survival of 5 and 24 hpf BAX mutant larvae expressing MOM-WT or MOM-ΔBH3-BAX. BAX mutant embryos expressing MOM-WT BAX shows similar mortality to those expressing untagged WT BAX from (A) ^∗∗∗^p < 0.001 (one-way ANOVA with Tukey’s post-test). Mean of 3 experiments (±SEM) is represented.

BAX is thought to participate in mitochondrion-mediated apoptosis through recruitment and oligomerization on the mitochondrial outer membrane to form pores (Dewson and Kluck, 2009). It has also been reported to target the mitochondrial outer membrane to mediate necrosis in conjunction with cyclophilin D in the inner mitochondrial membrane (Karch et al., 2013, Karch et al., 2015, Whelan et al., 2012). Different studies report this role as either dependent or independent of oligomerization, which requires its BH3 domain (Karch et al., 2013, Karch et al., 2015, Whelan et al., 2012) (Figure 2A). To test if BAX oligomerization was required in TNF-mediated necrosis, we expressed BAX lacking the BH3 domain (ΔBH3-BAX) in BAX-deficient animals (Figure 2A). We first confirmed the BH3 domain was required for apoptosis, as previously shown (Kratz et al., 2006): expression of full-length BAX in newly fertilized BAX-deficient zebrafish eggs was rapidly lethal (due to exacerbated apoptosis), whereas ΔBH3-BAX expression was not (Figure S2A). BAX mutants expressing ΔBH3-BAX also remained resistant to camptothecin-mediated apoptosis (Figure S2B). In contrast, ΔBH3-BAX restored TNF-mediated necrosis in BAX mutants, showing that BAX oligomerization and pore formation were not required (Figure 2B). Furthermore, BCB (BAX channel blocker), a small molecule inhibitor of BAX channel forming activity and cytochrome C release required for apoptosis (Cui et al., 2017), inhibited camptothecin-mediated apoptosis but not macrophage necrosis (Figures 2C and S2C). These results show that BAX functions in TNF-mediated necrosis independent of its oligomerization and pore-forming activity.

### BAX’s Role in TNF-Mediated Necrosis Does Not Require Its Localization to the Outer Mitochondrial Membrane

Prior reports described a necrosis pathway where non-oligomerizing BAX interacts with the mitochondrial outer membrane to mediate necrosis in conjunction with cyclophilin D acting in the inner membrane (Karch et al., 2013, Whelan et al., 2012). If this occurs in TNF-mediated necrosis, then expression of ΔBH3-BAX targeted to the mitochondrial outer membrane should be sufficient to restore necrosis in BAX-deficient animals. Strikingly, it did not (Figure 2D). We confirmed that the mitochondrial tag did not disrupt BAX activity by showing that the apoptotic function of mitochondrially tagged full-length BAX was intact. Expression of the construct was lethal to newly fertilized BAX-deficient eggs (Figure S2D). Therefore, BAX’s role in TNF-mediated cyclophilin D-dependent necrosis is not through targeting the mitochondrial outer membrane.

### BAX Mediates Necrosis by Promoting Ca^2+^ Translocation from the ER to the Mitochondrion

How might BAX facilitate cyclophilin D-dependent necrosis? In addition to its mitochondrial interactions, BAX is reported to regulate Ca^2+^ release from the ER (Rong and Distelhorst, 2008). Because mitochondrial Ca^2+^ overload, as well as oxidative stress, is known to be a trigger for cyclophilin D-dependent necrosis (Clarke et al., 2002, Halestrap, 2005), we wondered if BAX worked in TNF-mediated necrosis by facilitating Ca^2+^ flow from the ER to the mitochondrion to cause this overload. This model leads to five predictions: (1) TNF-mediated necrosis should be associated with mitochondrial Ca^2+^overload, (2) inhibiting mitochondrial Ca^2+^ overload should rescue necrosis, (3) mitochondrial Ca^2+^overload should be BAX-dependent as well as dependent on its upstream activators, (4) BAX’s action in the ER should be sufficient for mitochondrial Ca^2+^overload and necrosis, and (5) reducing ER Ca^2+^ stores should prevent necrosis.

We used two approaches to test if mitochondrial Ca^2+^overload was linked to necrosis. First, we visualized mitochondrial Ca^2+^ in animals expressing the genetically encoded Ca^2+^ indicator GCaMP3 specifically in mitochondria (Esterberg et al., 2014). TNF increased the intensity of GCaMP3 fluorescence only in infected macrophages within 90 min, indicating that it promoted mitochondrial Ca^2+^ uptake (Figures 2E and 2F), consistent with TNF causing necrosis specifically in infected macrophages (Roca and Ramakrishnan, 2013). In a complementary approach, we used Rhod-2, a fluorescent chemical probe for Ca^2+^ that accumulates in mitochondria (Smolina et al., 2014). Again infected, but not uninfected, macrophages became Rhod-2-positive within 120 min of TNF administration (Figures 2G and 2H). Mitochondrial Ca^2+^ overload in infected macrophages was rapidly followed by necrosis; in contrast, infected macrophages without Ca^2+^ overload did not die (Figure 2I). Thus, TNF caused mitochondrial Ca^2+^ overload selectively in infected macrophages and mitochondrial Ca^2+^ overload preceded necrosis.

**Figure 3:**
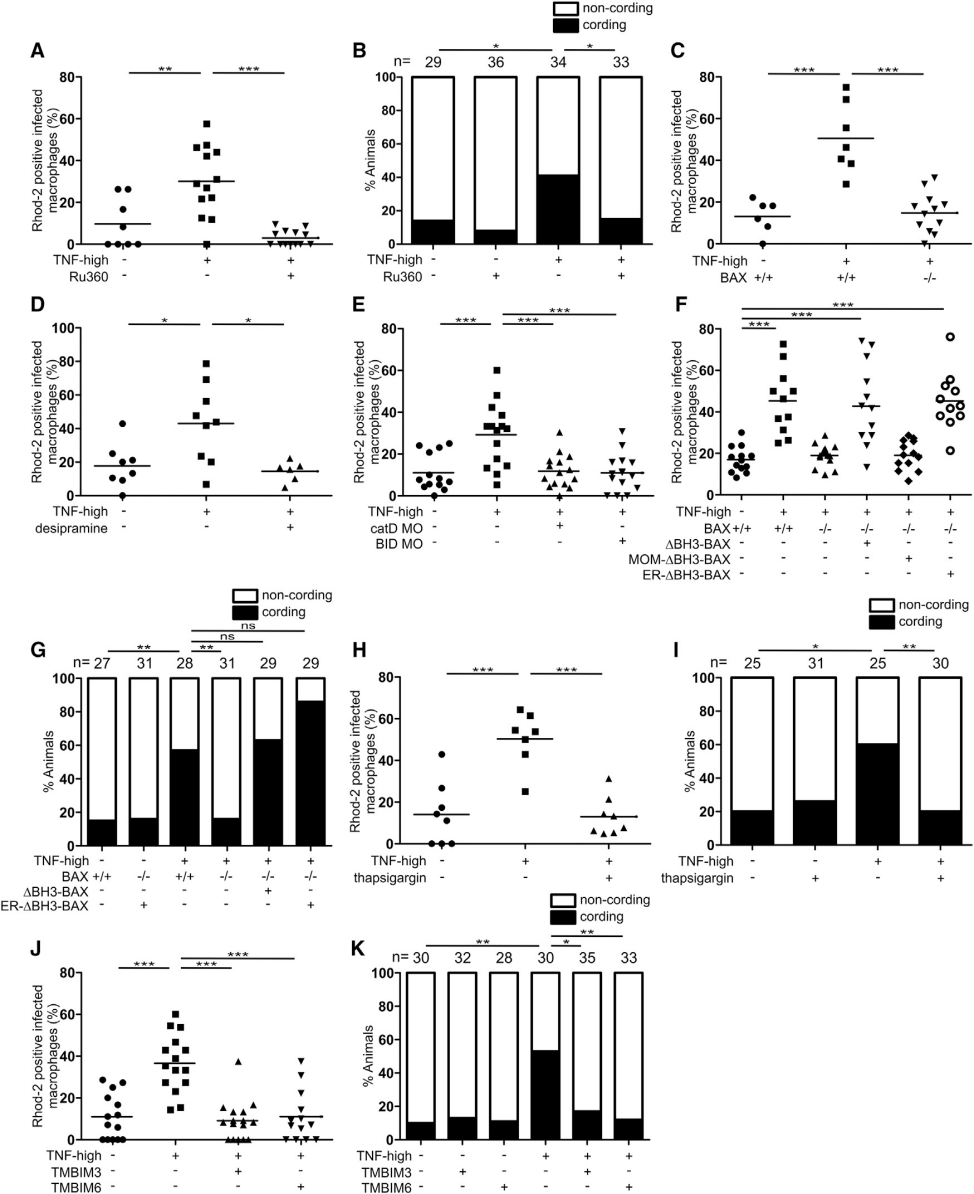
BAX Promotes Ca^2+^ Flow from the ER into the Mitochondrion (A) Percentage of Rhod-2-positive macrophages in 1 dpi control and TNF-high larvae treated with Ru360. Horizontal bars, means; ^∗∗^p < 0.01; ^∗∗∗^p < 0.001 (one-way ANOVA with Tukey’s post-test). Representative of 2 independent experiments. (B) Cording in 5 dpi TNF-high and control larvae treated with Ru360. ^∗^p < 0.05 (Fisher’s exact test). Representative of 5 independent experiments. (C) Percentage of Rhod-2-positive macrophages in 1 dpi control and TNF-high larvae that are WT or BAX mutant. Horizontal bars, means; ^∗∗∗^p < 0.001 (one-way ANOVA with Tukey’s post-test). Representative of 2 independent experiments. (D) Percentage of Rhod-2-positive macrophages in 1 dpi control and TNF-high larvae treated with desipramine. Horizontal bars, means; ^∗^p < 0.05 (one-way ANOVA with Tukey’s post-test). (E) Percentage of Rhod-2-positive macrophages in 1 dpi control and TNF-high larvae that are WT, cathepsin D, or BID morphant. Horizontal bars, means; ^∗∗∗^p < 0.001 (one-way ANOVA with Tukey’s post-test). Representative of 2 independent experiments. (F) Percentage of Rhod-2-positive macrophages in 1 dpi control and TNF-high larvae that are WT, BAX mutant, or BAX mutant expressing ΔBH3-BAX, MOM- or ER-targeted ΔBH3-BAX. Horizontal bars, means; ^∗∗∗^p < 0.001 (one-way ANOVA with Tukey’s post-test). Representative of 2 independent experiments. (G) Cording in 5 dpi TNF-high or control larvae that are WT, BAX mutant, BAX mutant expressing ΔBH3-BAX, or BAX mutant expressing ER-targeted ΔBH3-BAX. ^∗∗^p < 0.01 (Fisher’s exact test). Representative of 2 independent experiments. (H) Percentage of Rhod-2-positive macrophages in 1 dpi control and TNF-high larvae treated with thapsigargin. Horizontal bars, means; ^∗∗∗^p < 0.001 (one-way ANOVA with Tukey’s post-test). (I) Cording in 5 dpi TNF-high or control larvae treated with thapsigargin. ^∗^p < 0.05; ^∗∗^p < 0.01 (Fisher’s exact test). Representative of 3 independent experiments. (J) Percentage of Rhod-2-positive macrophages in 1 dpi control and TNF-high larvae that are WT or overexpressing TMBIM3 or TMBIM6. Horizontal bars, means; ^∗∗∗^p < 0.001 (one-way ANOVA with Tukey’s post-test). Representative of 2 independent experiments. (K) Cording in 5 dpi TNF-high or control larvae that are WT or overexpressing TMBIM3 or TMBIM6. ^∗^p < 0.05; ^∗∗^p < 0.01 (Fisher’s exact test). Representative of 2 independent experiments.

To test if inhibiting mitochondrial Ca^2+^ overload rescues necrosis, we treated animals with Ru360, an inhibitor of the mitochondrial Ca^2+^ uniporter (MCU), the major route of Ca^2+^ entry into the mitochondrial matrix (Zazueta et al., 1999). Ru360 prevented the Ca^2+^ overload seen in TNF-high animals at 5 h (Figure 3A) and inhibited macrophage necrosis at 5 dpi (Figure 3B). Thus, Ca^2+^ overload is a prerequisite for macrophage necrosis. Mitochondrial Ca^2+^ overload was absent in BAX-, ceramide-, cathepsin D-, and BID-deficient animals (Figures 3C–3E), indicating that BAX and its upstream activators were required for mitochondrial Ca^2+^ overload. To test if BAX causes mitochondrial Ca^2+^ overload solely by acting in the ER, we expressed ER-targeted ΔBH3-BAX in BAX-deficient animals. ER-targeted ΔBH3-BAX restored TNF-mediated mitochondrial Ca^2+^ overload and macrophage necrosis in BAX-deficient animals, similar to untargeted ΔBH3-BAX (Figures 3F and 3G).

To test if reducing ER Ca^2+^ prevents TNF-mediated necrosis, we used two approaches. First, we used thapsigargin to inhibit activity of ER membrane-resident SERCA proteins that transfer Ca^2+^ from the cytosol into the ER lumen (Chemaly et al., 2018); thapsigargin depletes ER Ca^2+^ while increasing cytosolic Ca^2+^ (Inesi and Sagara, 1992, Iwasaki et al., 2015). Thapsigargin treatment reduced macrophage mitochondrial Ca^2+^ overload and reduced necrosis (Figures 3H and 3I). Second, we overexpressed the ER Ca^2+^ leak channels TMBIM 3 and 6 in the animals to reduce steady-state ER Ca^2+^ concentrations (Lisak et al., 2015). Overexpression of either TMBIM 3 or 6 inhibited TNF-mediated mitochondrial Ca^2+^ overload and macrophage necrosis (Figures 3J and 3K). These findings revealed the ER, not the mitochondrion, is the target of activated BAX in TNF-mediated necrosis of Mm-infected macrophages, and BAX activation is required for Ca^2+^ translocation from the ER to the mitochondrion.

### BAX Mediates Direct Ca^2+^ Flow from the ER to the Mitochondrion through Ryanodine Receptors

**Figure 4:**
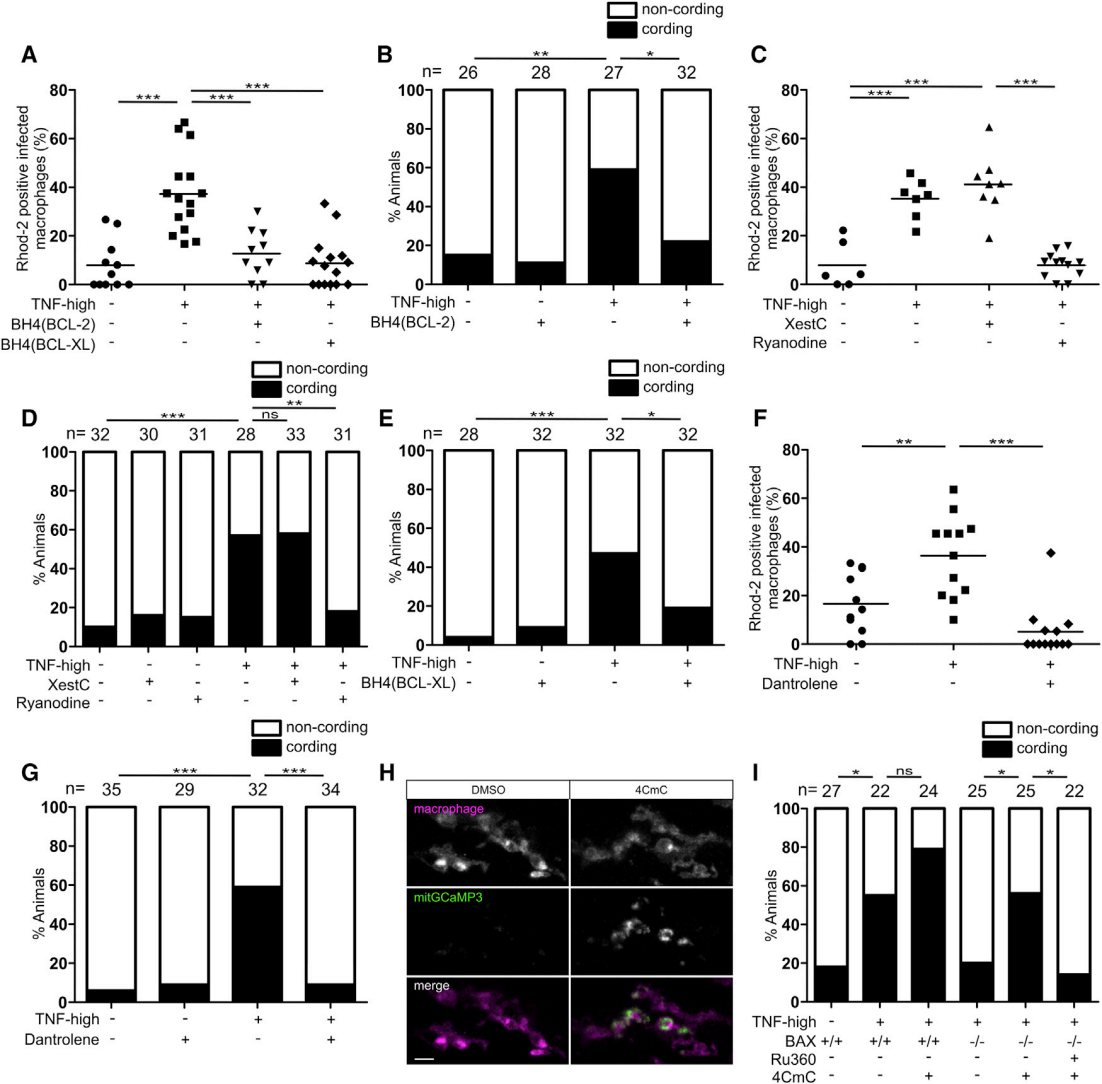
Ryanodine Receptor Mediates Macrophage Necrosis in Excess TNF Conditions (A) Percentage of Rhod-2-positive macrophages in 1 dpi control and TNF-high larvae that are WT or expressing the BH4 domain of BCL-2 or BCL-XL. Horizontal bars, means; ^∗∗∗^p < 0.001 (one-way ANOVA with Tukey’s post-test). (B) Cording in 5 dpi TNF-high and control larvae that are WT or expressing the BH4 domain of BCL-2. ^∗^p < 0.05; ^∗∗^p < 0.01 (Fisher’s exact test). Representative of 3 independent experiments. (C) Percentage of Rhod-2-positive macrophages in 1 dpi control and TNF-high larvae treated with xestospongin C (XestC) or ryanodine. Horizontal bars, means; ^∗∗∗^p < 0.001 (one-way ANOVA with Tukey’s post-test). Representative of 2 independent experiments. (D) Cording in 5 dpi TNF-high and control larvae treated with XestC or ryanodine. ^∗∗^p < 0.01; ^∗∗∗^p < 0.001 (Fisher’s exact test). Representative of 2 independent experiments. (E) Percentage of larvae with cording among WT or siblings expressing the BH4 domain of BCL-XL injected with TNF. ^∗^p < 0.05; ^∗∗∗^p < 0.001 (Fisher’s exact test). Representative of 2 independent experiments. (F) Percentage of Rhod-2-positive macrophages in 1 dpi control and TNF-high larvae treated with dantrolene. Horizontal bars, means; ^∗∗^p < 0.01; ^∗∗∗^p < 0.001 (one-way ANOVA with Tukey’s post-test). (G) Cording in 5 dpi TNF-high and control larvae treated with dantrolene. ^∗∗∗^p < 0.001 (Fisher’s exact test). Representative of 4 independent experiments. (H) Representative confocal images of 3 day larvae expressing GCaMP3 targeted to the mitochondria of their macrophages which are red fluorescent, 2 h after administration of 4CmC larvae. Scale bar, 10 μm. (I) Cording in 5 dpi TNF-high and control larvae that are WT or BAX mutant and treated with Ru360 and/or 4CmC. ^∗^p < 0.05 (Fisher’s exact test). Representative of 2 independent experiments. See also Table S1.

We searched for mechanisms by which BAX causes ER to mitochondrion Ca^2+^ translocation with resultant mitochondrial Ca^2+^ overload. Apoptosis can feature ER to mitochondrion Ca^2+^ translocation through the IP3 receptor (IP3R) (Jayaraman and Marks, 1997, Vance, 2014). To test if BAX was promoting mitochondrial Ca^2+^ transit through IP3R, we took advantage of the finding that BCL2 binds and inhibits IP3R through its BH4 domain (Rong et al., 2009). Expression of the 28-amino acid BCL2 BH4 domain inhibited mitochondrial Ca^2+^ overload and necrosis, suggesting IP3R involvement (Figures 4A and 4B). Surprisingly, xestospongin C, a specific IP3R inhibitor, which is active in the zebrafish (Esterberg et al., 2014), did not inhibit mitochondrial Ca^2+^ overload and necrosis (Figures 4C and 4D). In reconciling the disparity between the xestospongin C and BCL2 BH4 effects, we realized that BCL2 BH4 also inhibits another group of ER Ca^2+^ channels, the ryanodine receptors (RyR) (Vervliet et al., 2014, Vervliet et al., 2016). While RyR activity is recognized in excitable cell types, they are expressed in both human and zebrafish monocytes/macrophages (Table S1). To ask if RyR were involved in this pathway, we took advantage of the finding that only RyR, and not IP3R, are inhibited by the BH4 domain of BCL-XL (Vervliet et al., 2015). BCL-XL BH4 inhibited mitochondrial Ca^2+^ overload and necrosis similar to BCL-2 BH4, suggesting RyR involvement (Figures 4A and 4E). This was confirmed by showing that the specific RyR inhibitor ryanodine inhibited TNF-mediated mitochondrial Ca^2+^ overload and macrophage necrosis (Figures 4C and 4D). Another specific RyR inhibitor, dantrolene, an approved human drug, did as well (Zhao et al., 2001) (Figure 4F and 4G). Conversely, we asked if pharmacological activation of RyR with 4CmC (Jacobson et al., 2006) restored TNF-mediated necrosis in BAX mutants. We first confirmed 4CmC activity in the zebrafish by showing that it increased mitochondrial Ca^2+^ (Figure 4H). 4CmC did restore TNF-mediated necrosis in BAX mutants, and this was blocked by inhibiting mitochondrial Ca^2+^ uptake with Ru360 (Figure 4I). Thus, BAX acts at the ER through RyR to cause mitochondrial Ca^2+^ overload and necrosis.

### TNF-Induced Mitochondrial ROS Cause Activation of Lysosomal aSM that Ultimately Results in Mitochondrial Ca^2+^ Overload

**Figure S3:**
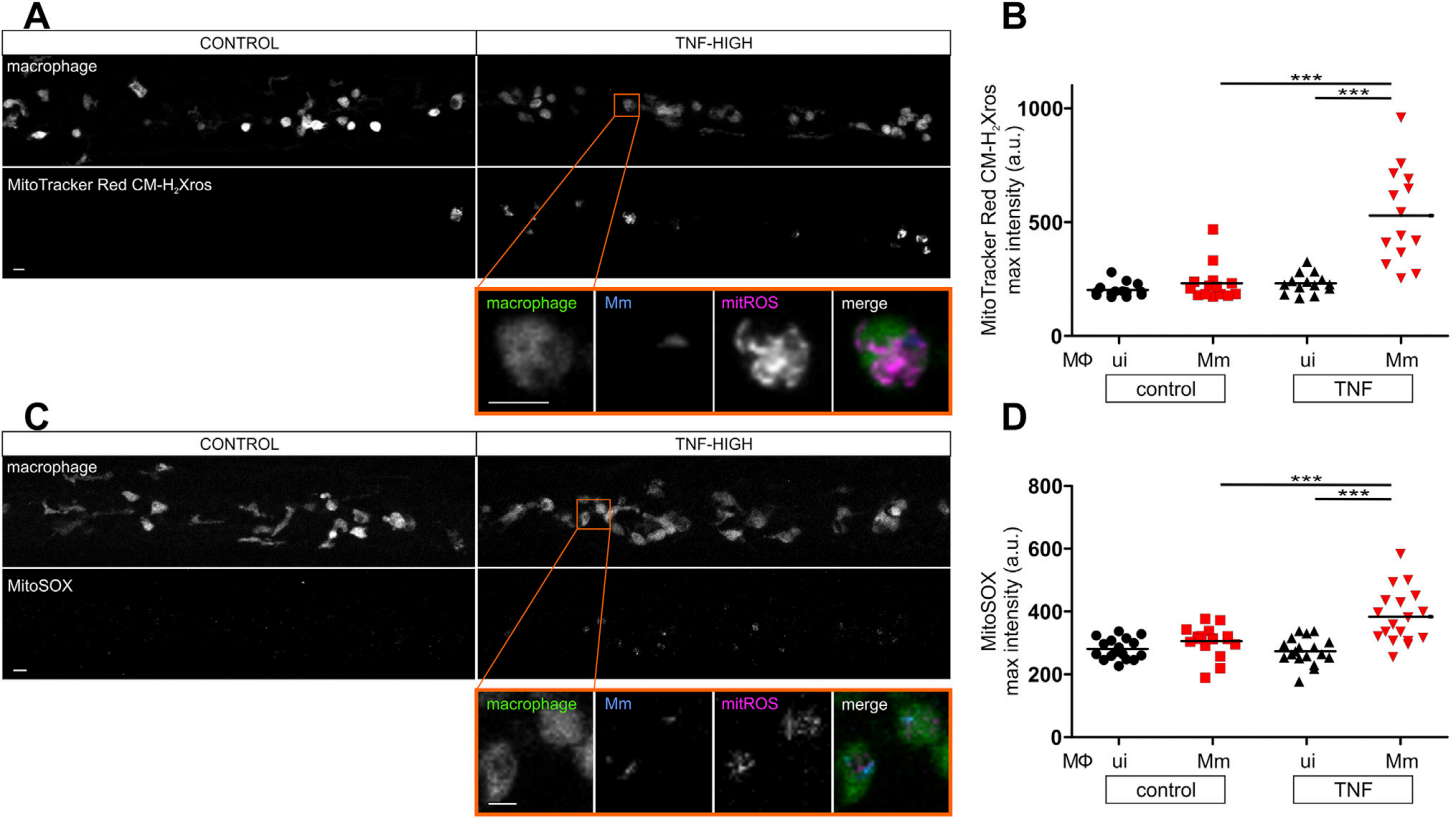
TNF Triggers Mitochondrial ROS Production Only in Infected Macrophages, Related to Figure 5 (A) Representative confocal images of 1 dpi TNF-high or control larvae with yellow fluorescent macrophages, showing MitoTracker Red CM-H_2_Xros fluorescence corresponding to similar area of the fish as in Figure 3A. Detail: macrophage in the orange rectangle. Scale bar 10 μm. (B) Quantification of mitochondrial ROS production in 1 dpi TNF-high or control larvae. Each point represents the mean of maximum intensity fluorescence of MitoTracker Red CM-H_2_Xros per fish from images in (A). Black and red symbols represent uninfected and Mm-infected macrophages, respectively, in the same control or TNF-administered animal. Horizontal bars, means; ^∗∗∗^p < 0.001 (one-way ANOVA with Tukey’s post-test). (C) Representative confocal images of 1 dpi TNF-high or control larvae showing MitoSOX fluorescence (red) corresponding to similar area of the fish as in Figure 3A. Detail: macrophage in the orange rectangle. Scale bar 10 μm. (D) Quantification of mitochondrial ROS production in 1 dpi TNF-high or control larvae. Each point represents the mean of maximum intensity fluorescence of MitoSOX per fish from images in (C). Black and red symbols represent uninfected and Mm-infected macrophages, respectively, in the same control or TNF-administered animal. Horizontal bars, means; ^∗∗∗^p < 0.001 (one-way ANOVA with Tukey’s post-test).

**Figure 5:**
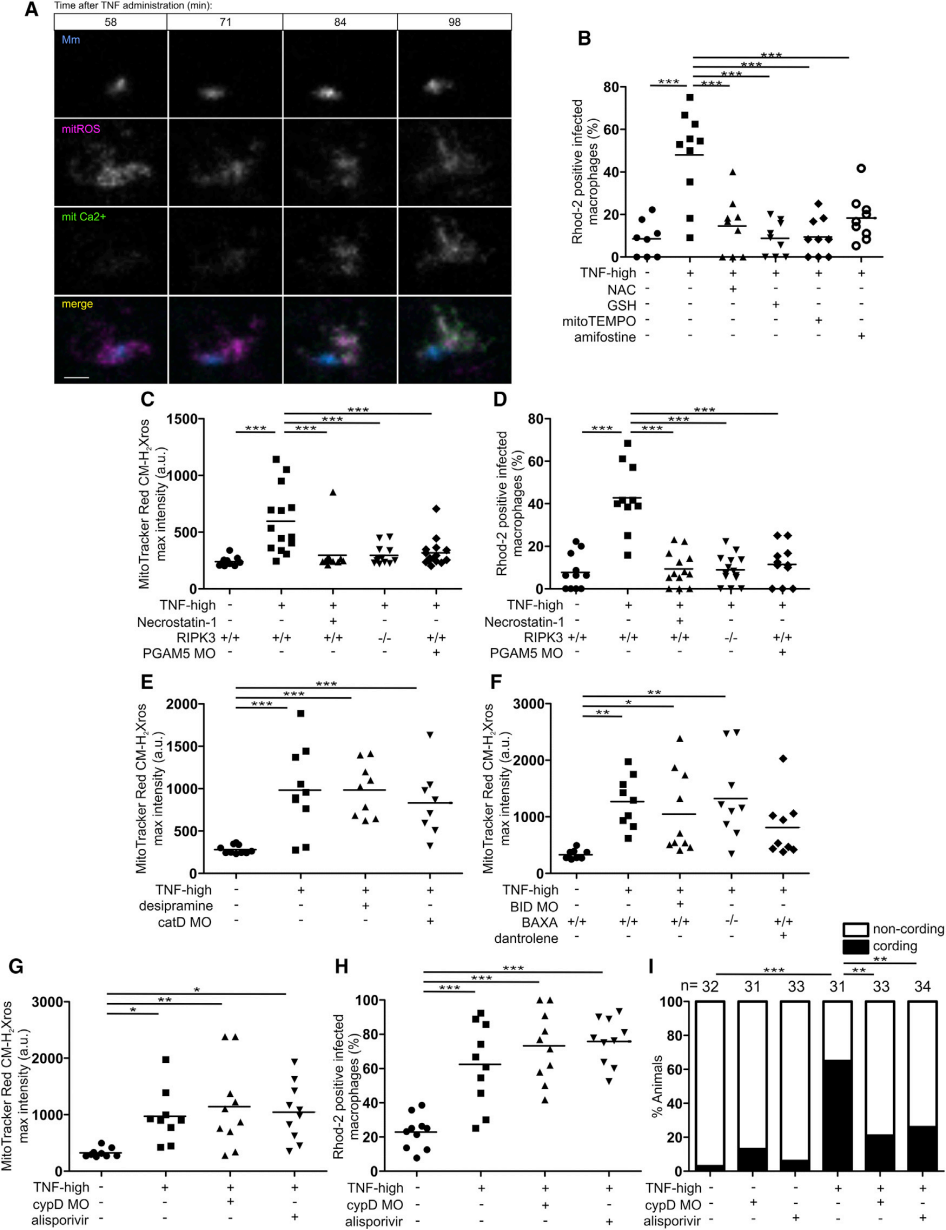
Mitochondrial ROS Launch the Pathogenic Mitochondrial-Lysosomal-ER Circuit that Leads to Mitochondrial Ca^2+^ Overload and Macrophage Necrosis (A) Time-lapse confocal images of an infected macrophage in a 1 dpi mitGCaMP3 larva at indicated time points after TNF administration with MitoTracker Red CM-H_2_Xros. Scale bar, 5 μm. (B) Percentage of Rhod-2-positive macrophages in 1 dpi control and TNF-high larvae treated with NAC, GSH, MitoTEMPO, or amifostine. Horizontal bars, means; ^∗∗∗^p < 0.001 (one-way ANOVA with Tukey’s post-test). (C) Quantification of mitochondrial ROS production in 1 dpi TNF-high or control larvae that are WT, WT treated with necrostatin-1, PGAM5 morphant, or RIPK3 mutant. Horizontal bars, means; ^∗∗∗^p < 0.001 (one-way ANOVA with Tukey’s post-test). (D) Percentage of Rhod-2-positive macrophages in 1 dpi control and TNF-high larvae control, that are WT, WT treated with necrostatin-1, PGAM5 morphant, or RIPK3 mutant. Horizontal bars, means; ^∗∗∗^p < 0.001 (one-way ANOVA with Tukey’s post-test). (E) Quantification of mitochondrial ROS production in 1 dpi TNF-high or control larvae that are WT, WT treated with desipramine, or cathepsin D morphant. Horizontal bars, means; ^∗∗∗^p < 0.001 (one-way ANOVA with Tukey’s post-test). (F) Quantification of mitochondrial ROS production in 1 dpi TNF-high or control larvae that are WT, WT treated with dantrolene, BID morphant, or BAX mutant. Horizontal bars, means; ^∗^p < 0.05; ^∗∗^p < 0.01 (one-way ANOVA with Tukey’s post-test). (G) Quantification of mitochondrial ROS production in 1 dpi TNF-high or control larvae that are WT, WT treated with alisporivir, or cyclophilin D morphant. Horizontal bars, means; ^∗^p < 0.05; ^∗∗^p < 0.01 (one-way ANOVA with Tukey’s post-test). (H) Percentage of Rhod-2-positive macrophages in 1 dpi TNF-high or control larvae that are WT, WT treated with alisporivir, or cyclophilin D morphant. Horizontal bars, means; ^∗∗∗^p < 0.001 (Fisher’s exact test). (I) Cording in 5 dpi TNF-high or control larvae that are WT, WT treated with alisporivir, or cyclophilin D morphant. ^∗∗^p < 0.01; ^∗∗∗^p < 0.001 (Fisher’s exact test). See also Figure S3.

We now understood that the lysosomal pathway ultimately converged into the mitochondrion to cause cyclophilin D-dependent necrosis. But how does TNF trigger lysosomal ceramide production in infected macrophages? Because aSM has been shown to be a redox sensitive enzyme (Dumitru and Gulbins, 2006), we hypothesized that TNF first induces mitochondrial ROS, which activate lysosomal components that cause mitochondrial Ca^2+^ overload. If mitochondrial ROS are ultimately responsible for mitochondrial Ca^2+^ overload, then: (1) they should precede mitochondrial Ca^2+^ overload, (2) removing mitochondrial ROS should remove Ca^2+^ overload. Using two different fluorogenic probes to detect ROS specifically in the mitochondrion, MitoTracker Red CMH_2_Xros and MitoSOX, we found that mitochondrial ROS were induced within 40 min after TNF administration to the animals, the first time point that it was technically feasible to image them (Figures S3A–S3D). As expected, increased mitochondrial ROS production was seen only in infected macrophages (Roca and Ramakrishnan, 2013) (Figures S3B and S3D). Mitochondrial ROS appeared to precede mitochondrial Ca^2+^ overload, first apparent at 90 min post-TNF treatment (Figure 2I). We confirmed this by assessing them in the same experiment (Figure 5A). Mitochondrial ROS were robustly detected at 58 min, the first observation time point, and did not increase further over the 40-min observation period. Mitochondrial Ca^2+^ was first detected at 71 min, increasing further over the observation period. Having shown that mitochondrial ROS precede mitochondrial Ca^2+^ overload, we tested if removing mitochondrial ROS would remove Ca^2+^ overload. Multiple ROS scavengers previously shown to inhibit TNF-mediated necrosis, including the mitochondrion-specific antioxidant MitoTEMPO (Roca and Ramakrishnan, 2013), eliminated mitochondrial Ca^2+^ overload (Figure 5B). Moreover, RIPK1, RIPK3, and PGAM5, determinants shown to be downstream of TNF and required for mitochondrial ROS production (Roca and Ramakrishnan, 2013), were also required for mitochondrial Ca^2+^ overload—RIPK1 inhibition with necrostatin-1 and genetic inhibition of RIPK3 and PGAM5 removed both mitochondrial ROS (Figure 5C) and Ca^2+^ overload (Figure 5D). These results confirmed that mitochondrial ROS are required for Ca^+^ overload.

We confirmed the order of the pathway with genetic and pharmacological manipulations of TNF-high animals. Ceramide, cathepsin D, BID and BAX, and RyR, which were upstream of mitochondrial Ca^2+^ overload (Figures 3C–3E, 4C, and 4F), were found to be downstream of mitochondrial ROS, as predicted—TNF-induced ROS were preserved upon aSM inhibition with desipramine, in cathepsin D and BID morphants, in BAX mutants, and upon RyR inhibition with dantrolene (Figures 5E and 5F).

Finally, our model predicts that cyclophilin D mediates necrosis downstream of both mitochondrial ROS and Ca^2+^ overload. To test this, we blocked cyclophilin D activity genetically and pharmacologically as previously shown (Roca and Ramakrishnan, 2013). Cyclophilin D morphants and alisporivir-treated animals preserved both mitochondrial ROS and Ca^2+^ overload (Figures 5G and 5H) and yet, as previously shown, did not manifest necrosis (Figure 5I). Together, these findings show that mitochondrial ROS initiate the circuit and confirm the predicted order of the components.

### Pharmacological Inhibition of Cellular Ca^2+^ Uptake Inhibits TNF-Mediated Macrophage Mitochondrial Ca^2+^ Overload and Necrosis in Mm- and Mtb-Infected Animals

**Figure 6:**
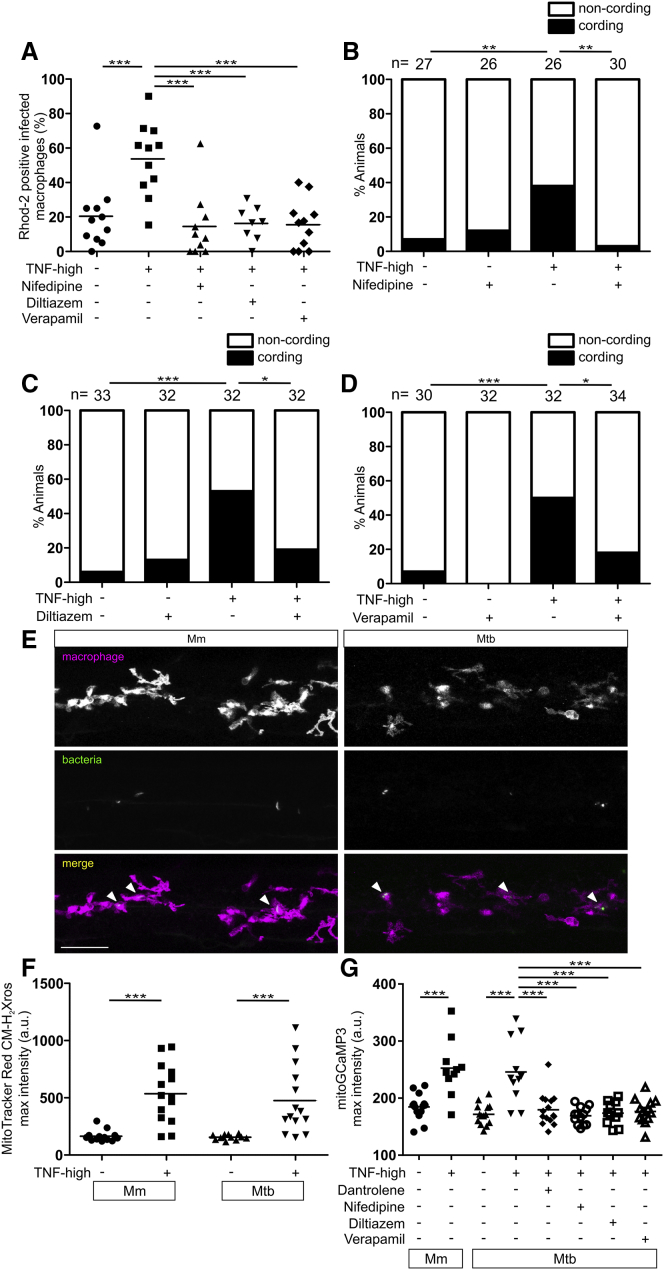
Pharmacological Inhibition of Voltage-Gated L-Type Ca^2+^ Channels Inhibits TNF-Mediated Macrophage Mitochondrial Ca^2+^ Overload and Necrosis in Mm- and Mtb-Infected Larvae (A) Percentage of Rhod-2-positive macrophages in 1 dpi control or TNF-high larvae treated with nifedipine, verapamil, or diltiazem. Horizontal bars, means; ^∗∗^p < 0.01; ^∗∗∗^p < 0.001 (one-way ANOVA with Tukey’s post-test). Representative of 2 independent experiments. (B–D) Cording in 5 dpi control or TNF-high larvae treated with nifedipine (B), diltiazem (C), and verapamil (D). ^∗^p < 0.05; ^∗∗^p < 0.01; ^∗∗∗^p < 0.001 (Fisher’s exact test). Representative of 2–4 independent experiments. (E) Representative confocal images of 1 dpi larvae with yellow fluorescent macrophages infected with 100 WT Mm or 80 Mtb (both red fluorescent). Scale bar, 50 μm. (F) Quantitation of mitochondrial ROS production in infected macrophages of 1 dpi TNF-high or control larvae infected with 100 WT Mm or 80 Mtb. Horizontal bars, means; ^∗∗∗^p < 0.001 (one-way ANOVA with Tukey’s post-test). (G) Quantitation of mitGCaMP3 fluorescence in infected macrophages in 1 dpi TNF-high or control larvae infected with 100 WT Mm or 185–200 Mtb treated with dantrolene, nifedipine, diltiazem, or verapamil. Horizontal bars, means; ^∗∗∗^p < 0.001 (one-way ANOVA with Tukey’s post-test).

Given the critical role of mitochondrial Ca^2+^ overload in necrosis, we wondered if reducing Ca^2+^ uptake in macrophages would lower steady-state ER Ca^2+^ levels sufficiently to prevent mitochondrial Ca^2+^ overload. Nifedipine, diltiazem, and verapamil represent different classes of drugs that inhibit voltage-gated L-type Ca^2+^ channels (LTCCs) located in the plasma membrane (Zamponi et al., 2015). While LTCCs were thought to be restricted to excitable cells, recent work has found them to be more broadly expressed, including in immune cells (Davenport et al., 2015, Espinosa-Parrilla et al., 2015). Importantly, LTCCs are present in human, mouse, and zebrafish myeloid cells, and modulate activation and cytokine production of human blood monocytes and alveolar macrophages (Antony et al., 2015, Athanasiadis et al., 2017, Ebina-Shibuya et al., 2017) (http://www.proteinatlas.org; www.immgen.org). We asked if nifedipine, diltiazem, and verapamil inhibit mitochondrial Ca^2+^ overload and macrophage necrosis in TNF-high animals and found that they did (Figures 6A–6D).

**Figure 7:**
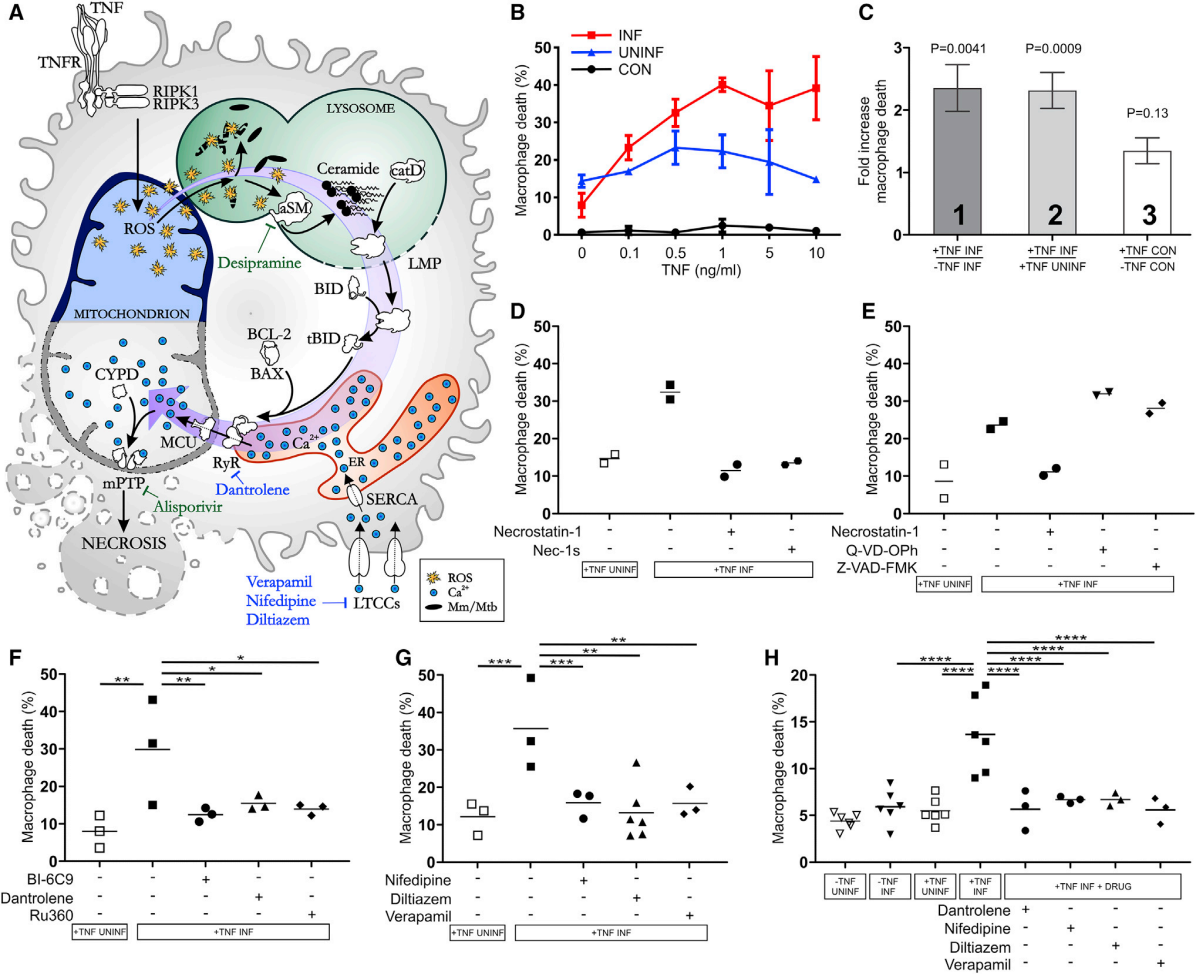
Mycobacterium-Infected Human Macrophages Undergo TNF-Mediated Necrosis (A) Model of TNF-mediated macrophage necrosis pathway. LMP, lysosomal membrane permeabilization; previously identified drugs in green, drugs identified in this study in blue. (B) THP-1 macrophage death 5 h after TNF administration (Mean ± SD). CON, macrophages from uninfected wells; INF, Mm-infected macrophages in infected wells; UNINF, uninfected macrophages in infected wells. (C) Quantification of TNF-induced macrophage death in multiple experiments. Column 1: ratio of TNF-treated to vehicle-treated dead Mm-infected macrophages. Column 2: ratio of dead infected macrophages to dead uninfected macrophages in the same TNF-treated well. Column 3: ratio of dead macrophages in TNF-treated to vehicle-treated uninfected wells. Mean ± SEM of 12 independent experiments for columns 1 and 2, and 11 independent experiments for column 3; one sample t test to a hypothetical value of 1, corresponding to the null hypothesis that TNF and infection do not influence cell death. (D) Percentage of dead Mm-infected macrophages after TNF administration treated with necrostatin-1 or Nec-1 s. Horizontal bars, means. Representative of 3 independent experiments. (E) Percentage of dead Mm-infected macrophages after TNF administration treated with necrostatin-1, Q-VD-OPh, or Z-VAD-FMK. Horizontal bars, means. Representative of 3 (necrostatin-1 and Z-VAD-FMK) or 2 (Q-VD-OPh) independent experiments. (F and G) Percentage of dead Mm-infected macrophages after TNF administration treated with BI-6C9, dantrolene, Ru360 (F), and diltiazem, nifedipine, or verapamil (G). Horizontal bars, means; ^∗^p < 0.05; ^∗∗^p < 0.01; ^∗∗∗^p < 0.001 (one-way ANOVA with Bonferroni’s post-test for comparisons shown). (H) Percentage of dead Mtb-infected macrophages after TNF administration treated with dantrolene, diltiazem, nifedipine, or verapamil. Horizontal bars, means; ^∗∗∗∗^p < 0.0001 (one-way ANOVA with Bonferroni’s post-test for comparisons shown). See also Figure S4 and Tables S1 and S2.

Our findings thus far showed this TNF-induced necrosis pathway is operant only in Mm-infected macrophages, with Mm required to induce mitochondrial ROS production in a macrophage-intrinsic fashion (Roca and Ramakrishnan, 2013) (Figures S3B and S3D). The therapeutic potential of the LTCC inhibitors in inhibiting pathogenic necrosis in human TB prompted us to check if Mtb similarly colludes with TNF to trigger this pathway. Virulent Mtb is a biosafety level 3 pathogen that we could not use in our biosafety level 2 zebrafish facilities. So we used the double leucine and pantothenate auxotroph of Mtb (Mtb Δ*leuD* Δ*panCD*), a biosafety level 2 pathogen owing to its inability to grow *in vivo* (Sampson et al., 2011). After confirming that Mtb Δ*leuD* Δ*panCD* persisted within zebrafish macrophages at 1 dpi (Figure 6E), we assessed early readouts of the pathway—macrophage mitochondrial ROS and mitochondrial Ca^2+^ overload. In agreement with the results from Mm infections, Mtb-infected macrophages of TNF-high animals displayed higher levels of mitochondrial ROS production and Ca^2+^ overload than infected macrophages in control animals (Figures 6F and 6G). Uninfected macrophages did not, as expected (data not shown). Importantly, the RyR inhibitor dantrolene and the LTCC inhibitors removed the mitochondrial Ca^2+^ overload from both Mtb and Mm (Figure 6G). In sum, Mtb, like Mm, co-opts host TNF to mediate macrophage necrosis, and dantrolene and the LTCC targeting drugs inhibit this pathogenic event, whether initiated by Mm or Mtb (Figure 7A).

### The TNF-Mediated Necrosis Pathway Occurs in Mycobacterium-Infected Human Macrophages

If Mtb, a human pathogen, induces the necrosis pathway in the zebrafish, then the pathway is likely to be operant in human macrophages. To test this, we used macrophages derived from the human monocytic cell line THP-1, which express RyR and LTCCs (Table S1) (http://www.proteinatlas.org). The zebrafish findings lead to four testable predictions for human macrophages: (1) TNF should increase death of infected but not uninfected macrophages, (2) TNF-induced death should occur through RIPK1-mediated necrosis (i.e., it is RIPK1-dependent and caspase-independent), (3) it should be dependent on BID, RyR, and mitochondrial Ca^2+^ overload, and (4) it should be inhibited by inhibiting Ca^2+^ uptake by the cells. To test these predictions, we infected THP-1 macrophages with Mm or Mtb for 24 h, then added exogenous TNF to the culture medium to create a TNF-high state. 5 h of TNF treatment increased cell death in a concentration-dependent fashion in Mm-infected macrophages; this was specific to infected cells and spared uninfected cells in the same well and cells in uninfected wells (Figure 7B). The highest TNF concentration tested, 10 ng/mL, gave the clearest separation between infected and uninfected cells, so we chose it for further experiments. A series of replicate experiments (Table S2) confirmed that TNF increased infected macrophage death (Figure 7C, column 1), in the TNF-treated wells, increased death was limited to infected macrophages (Figure 7C, column 2), and TNF did not increase macrophage death in uninfected wells (Figure 7C, column 3). Thus, we confirmed that TNF increases death of, and only of, Mm-infected macrophages.

**Figure S4:**
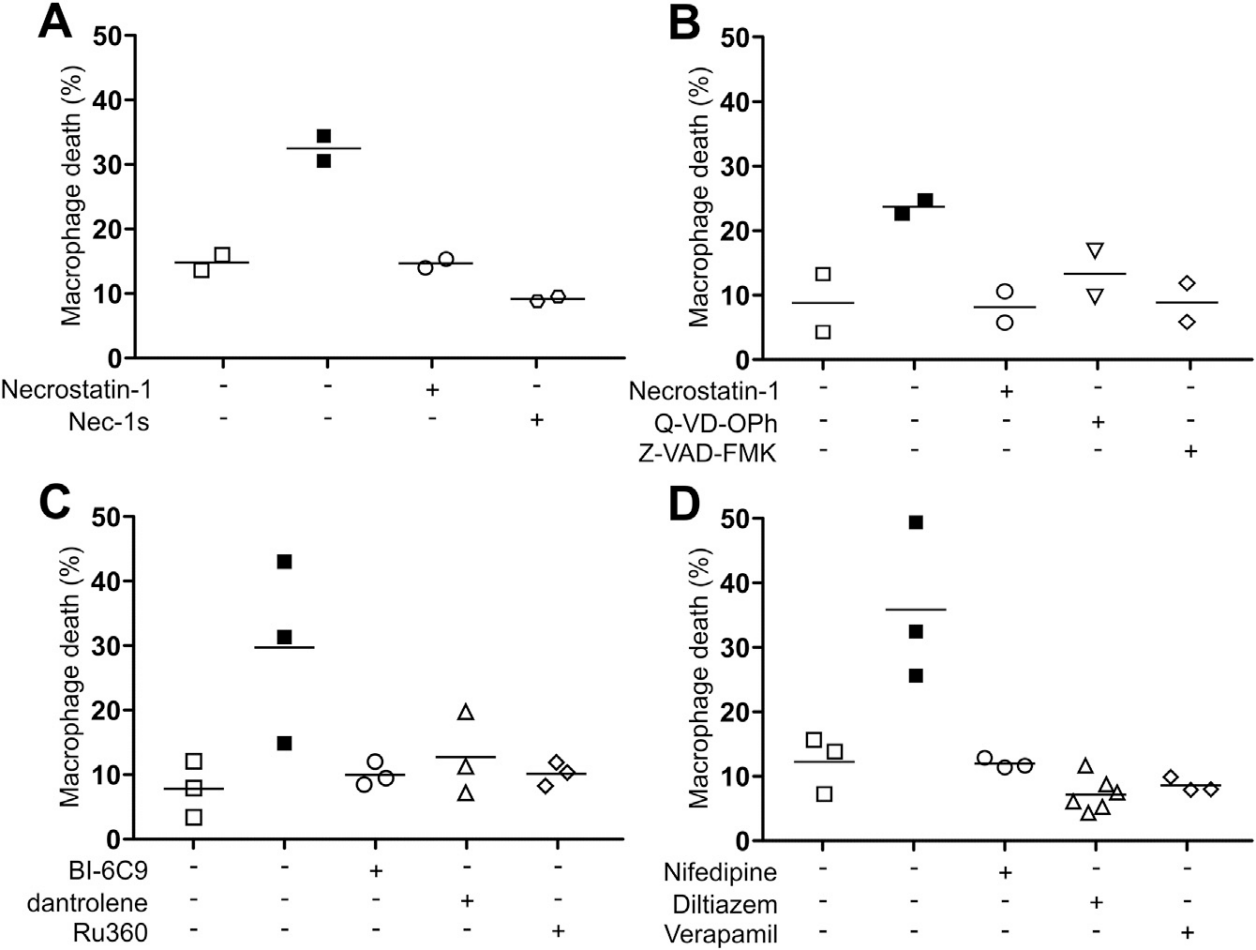
Treatment with Inhibitors of the TNF-Mediated Necrosis Pathway in the Presence of TNF Does Not Influence Death of Uninfected Macrophages, Related to Figure 7 (A) Percentage of dead Mm-infected (filled symbols) or uninfected macrophages after TNF administration treated with necrostatin-1 or nec-1 s. Horizontal bars, means. (B) Percentage of dead Mm-infected (filled symbols) or uninfected macrophages after TNF administration treated with necrostatin-1, Q-VD-OPh, or Z-VAD-FMK. Representative of 3 (necrostatin-1 and Z-VAD-FMK) or 2 (Q-VD-OPh) independent experiments. Horizontal bars, means. (C) Percentage of dead Mm-infected (filled symbols) or uninfected macrophages after TNF administration treated with BI-6C9, dantrolene or Ru360. Horizontal bars, means. (D) Percentage of dead Mm-infected (filled symbols) or uninfected macrophages after TNF administration treated with diltiazem, nifedipine or verapamil. Horizontal bars, means.

To test if infected macrophage death was RIPK1-dependent and caspase-independent, we used RIPK1 and pan-caspase inhibitors. The RIPK1 inhibitors necrostatin-1 and Nec-1s (Takahashi et al., 2012) inhibited TNF-mediated death whereas the pan-caspase inhibitors Q-VD-OPh and Z-VAD-FMK did not (Figures 7D and 7E). None of the inhibitors affected death of uninfected cells (Figures S4A and S4B). These results confirm that TNF mediates RIPK1-dependent necrosis selectively in infected human macrophages. Next, we inhibited BID, RyR, and MCU activity with BI-6C9, dantrolene, and Ru360. All inhibited infected, but not uninfected, macrophage necrosis showing the requirement for BID, RyR, and mitochondrial Ca^2+^ overload (Figures 7A, 7F, and S4C). We then used the LTCC inhibitors to ask if reducing Ca^2+^ uptake by the macrophages would inhibit necrosis. All three drugs inhibited necrosis specifically in infected cells (Figures 7G and S4D).

Finally, we confirmed that Mtb-infected human macrophages are susceptible to TNF-mediated necrosis. TNF increased necrosis of infected but not uninfected macrophages and was inhibited by dantrolene and the LTCC inhibitors (Figure 7H). As with Mm, inhibition of death by dantrolene and the LTCC inhibitors was specific to infected cells, sparing uninfected cells (Figure S4E). In sum, both Mm and Mtb co-opt TNF to mediate necrosis in human macrophages.

## Discussion

Our dissection of the mechanism by which an excessive TNF response causes necrosis of mycobacterium-infected macrophages has revealed an intricate inter-organellar relay—a circuit that begins in the mitochondrion, transits the lysosome, cytosol, and ER to complete in the mitochondrion (Figure 7A). Active in both zebrafish and human macrophages, this necrosis pathway exemplifies important signaling circuits conserved from fish to humans. Given the shared pathogenetic programs of Mm and Mtb, particularly in the context of mycobacterium-macrophage interactions and the tuberculous granuloma (Cambier et al., 2014), it is not surprising that signaling networks uncovered in the context of fish TB are conserved in human TB. The use of the zebrafish has uncovered this conserved pathogenic necrosis program and suggested drugs to inhibit it (Figure 7A). Prior work identified the aSM-inhibiting tricyclic antidepressant drug, desipramine, and the CYPD inhibitor, alisporivir, to inhibit this necrosis (Roca and Ramakrishnan, 2013) (Figure 7A). The more complete understanding of the program suggests dantrolene, and most promisingly, the widely used, inexpensive LTCC inhibitors as TB treatments (Figure 7A). These host-targeting drugs should be effective in drug-sensitive and drug-resistant TB.

Our work assigns new functions to, and links between, several participants that execute TNF-mediated necrosis in mycobacterium-infected macrophages. We identify BAX as an activator of RyR. RyR are Ca^2+^ translocators enabling activity of excitable tissues; we find macrophage activity of these receptors mediates pathology in mycobacterial infection. Our dissection of the pathway reveals unexpected interconnections between effector molecules that span different organelles. The circuit begins and ends with the transit of two inorganic signals—ROS from mitochondrion to lysosome and Ca^2+^ from ER to mitochondrion—and requires cathepsin D translocation from lysosome to cytosol.

This pathway does not fit into current classification schemes of regulated cell death (Galluzzi et al., 2018), being dependent on RIPK1, RIPK3, and possibly MLKL, similar to necroptosis, and on CYPD, similar to CYPD-mediated necrosis. Further confounding current categorization, the pathway is dependent on activity of lysosomal cathepsin D activity in the cytosol. Yet, it does not display the generalized lysosomal permeabilization that demarcates another discrete cell death category, lysosome-dependent cell death (Galluzzi et al., 2018). An important player is the mycobacterium whose presence in the macrophage is required for mitochondrial ROS production in response to excess TNF (Roca and Ramakrishnan, 2013). Whether infection has further roles in executing this necrosis remains to be determined. What is clear is that mycobacterium-infected macrophages undergo a specific form of regulated death involving multiple organelles and a high degree of cross-talk between components associated with different cell death programs (Galluzzi et al., 2018). The idea that necrotic cell death can occur through the interplay between multiple signaling pathways rather than a single well-described one is not new (Festjens et al., 2006, Vanden Berghe et al., 2014). This work reveals that the dominant paradigms of cell death are insufficient to explain pathological macrophage death in TB.

### Inter-Organellar Contacts Promote Pathogenic Macrophage Necrosis

Ca^2+^ transfer from ER to mitochondrion is facilitated by physical contacts between them—the mitochondria-associated ER membranes (MAMs) (Naon and Scorrano, 2014, Vance, 2014). These tight connections allow for direct Ca^2+^ transit, creating a high Ca^2+^ concentration in the vicinity of the mitochondrial Ca^2+^ uniporter MCU, essential for Ca^2+^ transit through this low-affinity channel (Penna et al., 2018). Specifically, RyR-mediated ER-mitochondrial Ca^2+^ transfer is supported by findings showing that RyR activity is directly or indirectly coupled to VDAC, to promote Ca^2+^ translocation from the ER into the mitochondrion (Fernandez-Sanz et al., 2014, Min et al., 2012). Recent studies also find that mitochondrion-lysosome contacts occur at appreciable frequency in healthy cells, suggesting a mechanism by which mitochondrial ROS can transit to lysosomes (Valm et al., 2017, Wong et al., 2018) (Figure 7A). Our work implicates inter-organellar contacts, thought to be part of cellular homeostasis, in disease pathogenesis as well.

### Mitochondrial ROS Translocate Ca^2+^ into the Mitochondrion through an Elaborate Inter-organellar Circuit

Mitochondrial oxidative stress triggers mitochondrial transition pore opening by oxidizing and regulating cyclophilin D activity (Halestrap, 2005, López-Erauskin et al., 2012). Therefore, it was reasonable to think that TNF-mediated mitochondrial ROS induced necrosis by directly activating cyclophilin D (Roca and Ramakrishnan, 2013). Instead, we now find that ROS activate lysosomal components that ultimately deliver the Ca^2+^ into the mitochondrion that is essential for necrosis. These observations are in agreement with previous studies showing that Ca^2+^ regulates cyclophilin D activity and mPTP opening (Baines et al., 2005, Clarke et al., 2002, Nakagawa et al., 2005). While mitochondrial ROS may also directly regulate cyclophilin D in this pathway, our work shows it is unlikely their predominant role—interventions that remove mitochondrial Ca^2+^ uptake, while maintaining TNF-induced mitochondrial ROS, inhibit necrosis almost completely. Thus, mitochondrial ROS alone cannot mediate TNF-induced cyclophilin D-dependent necrosis. This work suggests the two inorganic signals co-operate to produce necrosis by each activating an enzyme—ROS activating the lysosomal enzyme aSM and Ca^2+^ activating the mitochondrial enzyme cyclophilin D.

### Extralysosomal Cathepsin D Function in TNF-Mediated Necrosis

The role assigned to cathepsin D in TNF-mediated necrosis—cleaving BID to its active state—raises the question of how a lysosomal protease makes its way to the cytosol. Lysosomal protease release from massive lysosomal disruption can cause necrosis (Turk et al., 2002). However, we can infer that cathepsin D works in TNF-mediated necrosis in the absence of massive lysosomal disruption as blocking the pathway immediately downstream of cathepsin D rescues necrosis. Our findings are most consistent with cytosolic translocation of cathepsin D through selective lysosomal membrane permeabilization (Ferri and Kroemer, 2001, Turk et al., 2002). Local increases in lysosomal ROS and sphingosine can produce lysosome selective disruptions, and oxidative stress is associated with cathepsin D cytosolic translocation (Ferri and Kroemer, 2001, Kågedal et al., 2001). Mitochondrial ROS that reach the lysosome may play the dual role of activating aSM and permeabilizing the lysosomal membrane, thus coordinating cathepsin D activation and release into the cytosol. Lysosomal ceramide is converted to sphingosine, thus overproduction of ceramide might also increase lysosomal sphingosine, which could further promote lysosomal permeabilization.

Cathepsin D is documented to function in the cytosol in multiple contexts (Benes et al., 2008). It can cleave BID at neutral pH *in vitro*, which explains its retaining activity in the cytosol (Appelqvist et al., 2012). Our findings do raise the question of why cathepsin D (and not cathepsin B) is specifically required in the pathway given that both cathepsins are activated by ceramide *in vitro* and are translocated in response to oxidative stress to the cytosol where both can cleave BID (Appelqvist et al., 2012, Heinrich et al., 2004, Kågedal et al., 2001). One possible explanation is that “emergency” cytosolic protease inhibitors (e.g., cystatins) can inhibit cysteine proteases like cathepsin B but no such inhibitors have yet been identified for the sole lysosomal aspartyl protease cathepsin D (Turk et al., 2002). Notably, earlier work on cathepsin D-deficient mice already highlighted the importance of cathepsin D’s extralysosomal function—cathepsin D-deficient mice were found to maintain normal bulk lysosomal proteolysis while undergoing widespread tissue destruction and early death (Saftig et al., 1995). The elaborate mechanisms that enable cathepsin D cytosolic activity and translocation are rooted in the requirement for its homeostatic proteolytic function. Our work now highlights that these same mechanisms in overdrive can enable TB pathogenesis.

### BAX as an Activator of RyR to Cause Ca^2+^ Translocation in Mycobacterium-Infected Macrophages

It is now appreciated that BAX, initially identified as a mediator of apoptosis, participates in necrosis and autophagy (Irrinki et al., 2011, Karch et al., 2015, Karch et al., 2017, Tischner et al., 2012, Whelan et al., 2012). In most cases, its function is disrupting organellar membranes—predominantly the mitochondrion but also ER and lysosome—generally through oligomerization to form pores or destabilizing membranes through other means (Feldstein et al., 2006, Karch et al., 2015, Karch et al., 2017, Whelan et al., 2012).

BAX is also implicated in maintaining ER Ca^2+^ levels, by sequestering BCL-2, which can reduce ER Ca^2+^ through interactions with multiple Ca^2+^ channels (Bonneau et al., 2013, Vervliet et al., 2016). We now find a new function for BAX as a translocator of Ca^2+^ from the ER to the mitochondrion by modulating RyR activation. Whether its action is direct or indirect remains to be determined, however, we know it occurs independently of its oligomerization and pore forming function.

The involvement of RyR rather than IP3R is another unexpected element. IP3R are ubiquitously present and trigger Ca^2+^ release in all cells; IP3R activity can promote apoptotic cell death by enabling mitochondrial Ca^2+^ overload through ER MAMs (Vervliet et al., 2016). We now find a similar role for RyR, which are thought to induce rapid translocation of Ca^2+^ in the context of nerve and muscle excitation (Lanner et al., 2010). Their expression in macrophages across species suggests that they must mediate Ca^2+^ transfer in nonexcitable cells as well, perhaps with different kinetics. Our findings highlight this function, albeit in a pathological context, and we show that dantrolene can inhibit its detrimental role.

### Dysregulated TNF as a General Cause of Tuberculous Granuloma Necrosis

Our quest to understand the detailed mechanism of TNF-mediated macrophage necrosis in the zebrafish was instigated by its potential relevance for TB severity and treatment in humans with a common genetic *LTA4H* variant that produces a hyperinflammatory state (Thuong et al., 2017, Tobin et al., 2012). However, it is likely that high LTA4H and TNF levels may be in play locally to produce granuloma necrosis even independently of *LTA4H* genotype. Spatial studies combining mass spectrometry with confocal microscopy of dissected human tuberculous granulomas show that necrotic tuberculous granulomas are enriched for LTA4H and TNF as compared to nonnecrotic granulomas from the same lung (Marakalala et al., 2016). Lactosylceramide, a downstream product of ceramide that is increased in cells by pro-inflammatory cytokines, is also increased in human lung necrotic granulomas (Chatterjee and Pandey, 2008, Kim et al., 2010). This lactosylceramide may reflect further metabolism of acid sphingomyelinase-derived ceramide in granuloma macrophages undergoing TNF-mediated necrosis. Thus, the TNF-initiated circuit we have identified may well play a wide role in tuberculous granuloma necrosis.

Deciphering TB pathogenesis has revealed new cell biology and signaling circuitry. Many noninfectious inflammatory conditions feature programmed necrosis that may be detrimental (Zhou and Yuan, 2014). It is conceivable that some are the result of the pathway we have identified, initiated by dysregulated TNF coupled to a second trigger to induce mitochondrial ROS. Finally, the identification of mitochondrial Ca^2+^ overload as a requisite for necrosis led us to ask if drugs that block LTCCs would reduce Ca^2+^ entry into the macrophage itself and thereby prevent the pathological mitochondrial overload required for necrosis. Our finding that a panel of widely used Ca^2+^ channel blocking drugs prevent both mitochondrial Ca^2+^ overload and necrosis suggests a potential for these drugs for TB sufferers with *LTA4H*-high genotype and possibly more generally for TB as well as other inflammatory necroses where this pathway might be operant. Finally, we note that the LTCC inhibitor verapamil also inhibits mycobacterial drug tolerance through its action on bacterial drug efflux pumps by a different mechanism (Adams et al., 2011, Adams et al., 2014, Gupta et al., 2013). In this light, verapamil holds particularly exciting prospects as an adjunctive agent in TB by dually blocking detrimental bacterial and host processes.

## STAR★Methods

### Key Resources Table

**Table:** 

REAGENT or RESOURCE	SOURCE	IDENTIFIER
**Bacterial and Virus Strains**		

*Mycobacterium marinum* M strain transformed with pMSP12:tdTomato	Takaki et al., 2013	derivatives of ATCC #BAA-535
*M. marinum* M strain transformed with pMSP12:EBFP2	Takaki et al., 2013	derivatives of ATCC #BAA-535
*M. marinum* M strain transformed with pMSP12:tdKatushka	Takaki et al., 2013	derivatives of ATCC #BAA-535
*M. marinum* M strain transformed with pMSP12:wasabi	Takaki et al., 2013	derivatives of ATCC #BAA-535
*M. tuberculosis* ΔleuD ΔpanCD double auxotroph strain transformed with pMSP12:mCherry	A. Floto Laboratory	N/A
*M. tuberculosis ΔleuDΔpanCD* double auxotroph strain transformed with pMSP12:GFP	A. Floto Laboratory	N/A

**Chemicals, Peptides, and Recombinant Proteins**		

Zebrafish recombinant TNF	Roca et al., 2008	N/A
Pepstatin A	Sigma-Aldrich	Cat#P4265; CAS: 26305-03-3
E64d	Sigma-Aldrich	Cat#E8640; CAS: 88321-09-9
BI-6C9	Sigma-Aldrich	Cat#B0186; CAS: 791835-21-7
(S)-(+)-Camptothecin (CPT)	Alfa Aesar	Cat#J62523; CAS: 7689-03-4
Acridine orange (2% solution in H_2_O)	Sigma-Aldrich	Cat#A9231; CAS: 65-61-2
BCB (Bax Channel Blocker)	Alfa Aesar	Cat#J64257
Rhod-2, AM, cell permeant	Fisher Scientific	Cat#R1245MP
Ru360	VWR International	Cat#557440
Amifostine	Cambridge Bioscience	Cat#14398-50mg-CAY
NAC (N-Acetyl-L-Cysteine)	Cambridge Bioscience	Cat#A0918-10 g
Mitotempo	Cambridge Bioscience	Cat#16621-5mg-CAY
GSH	Cambridge Bioscience	Cat#1242-1
Alisporivir	Novartis	Roca and Ramakrishnan, 2013
Xestospongin C (XestC)	Cambridge Bioscience	Cat#64950-10 ug-CAY
Ryanodine	Generon	Cat#2489-500
Dantrolene sodium salt	Sigma-Aldrich	Cat#D9175-1G; CAS: 14663-23-1
Nifedipine	Cambridge Bioscience	Cat#N3228-1 g
Diltiazem	Cambridge Bioscience	Cat#D3447-1 g
Verapamil HCl	Fisher Scientific	Cat#10403045
Thapsigargin	Sigma-Aldrich	Cat#T9033-.5MG; CAS: 67526-95-8
4-Chloro-*m*-cresol (4CmC)	Sigma-Aldrich	Cat#55402-5G; CAS: 59-50-7
MitoTracker Red CM-H_2_Xros	Fisher Scientific	Cat# M7513
MitoSOX	Fisher Scientific	Cat#M36008
Desipramine hydrochloride	Sigma-Aldrich	Cat#D3900-5G; CAS: 58-28-6
Necrostatin-1	Alfa Aesar	Cat#J65341.F+; CAS: 4311-88-0
Necrosulfonamide (NSA)	Cambridge Bioscience	Cat#20844-5mg-CAY; CAS: 1360614-45-7
PTU (1-phenyl-2-thiourea)	Sigma-Aldrich	Cat#P7629; CAS: 103-85-5
Tango Buffer (10x)	Thermo Scientific	Cat#BY5
PMA (Phorbol 12-myristate 13-acetate)	Sigma-Aldrich	Cat#P1585-1MG; CAS: 16561-29-8
Human recombinant TNF	Sigma-Aldrich	Cat#SRP3177-50UG
D (+) Trehalose dehydrate	Sigma-Aldrich	Cat#T0167-10G
Nec-1 s	Cambridge Bioscience	Cat#2263-5
Q-VD-OPh	Source Bioscience	Cat#ABE283
Z-VAD-FMK	Cambridge Bioscience	Cat#14463-1MG-CAY
SYTOX® Green Nucleic Acid Stain	Life Technologies	Cat#S7020

**Gibson Assembly**		

NEBuilder HiFi DNA Assembly Master Mix	New England Biolabs	Cat#E2621
RNeasy Mini Kit	QIAGEN	Cat#74104
mMessage mMachine kit	Ambion	N/A
polyA Tailing kit	Ambion	N/A

**Experimental Models: Cell Lines**		

THP-1 human monocytic cell line	ATCC	TIB-202

**Experimental Models: Organisms/Strains**		

Zebrafish (*Danio rerio*): wild type AB strain	University of Cambridge	ZFIN ID: ZDB-GENO-960809-7
Zebrafish: *Tg(mpeg1:YFP)*^*w200*^	Roca and Ramakrishnan, 2013	ZFIN ID: ZDB-FISH-150901-6828
Zebrafish: *Tg(mpeg1:Brainbow)*^*w201*^	Pagán et al., 2015	ZFIN ID: ZDB-FISH-151204-7
Zebrafish: *baxa*^*rr1*^	This work	N/A
Zebrafish: *baxb*^*rr10*^	This work	N/A
Zebrafish: *ripk3*^*rr7*^	This work	N/A
Zebrafish: *Tg(BH:GFP-mfap4:Mito-GCaMP3)*	This work	N/A

**Oligonucleotides**		

Morpholino: Cathepsin D E2/I2 TGTCAGCAA GCAGATACTCACATCT	Gene Tools; Follo et al., 2011	ZDB-MRPHLNO-110722-2
Morpholino: BID GGTCAAAGTTCCTGTTGAAGTCCAT	Gene Tools; Kratz et al., 2006	ZDB-MRPHLNO-070126-2
Morpholino: PGAM5 AGCGCCCTCCGAAAA GACATGCTTC	Roca and Ramakrishnan, 2013	ZDB-MRPHLNO-130820-4
Morpholino: cyclophilin D-ATG TTGGGTTTG ACATTTTCTTAGAT	Gene Tools; Roca and Ramakrishnan, 2013	ZDB-MRPHLNO-130820-5
Baxa^rr1^ forward primer for genotyping by HRM, sequence: 5′-GACCTCTGCCTCTTGCAGCTT-3′	This work	N/A
Baxa^rr1^ reverse primer for genotyping by HRM, sequence: 5′-GCAAACACTGCGCGAGGCGTT-3′	This work	N/A
Baxb^rr10^ forward primer for genotyping by HRM, sequence: 5′-GTTGCATCAAGTTTATGAGGTGTTG-3′	This work	N/A
Baxb^rr10^ reverse primer for genotyping by HRM, sequence: 5′-CAGGGTAGTAAGGCAGGAG CTAATGG-3′	This work	N/A
RIPK3^rr7^ forward primer for genotyping by HRM, sequence: 5′-AGAGAAGCGGAGCTGATGTTTGAT-3′	This work	N/A
RIPK3^rr7^ reverse primer for genotyping by HRM, sequence: 5′-CTTGGCATCCAGGCTGTCACTCAGC-3′	This work	N/A

**Recombinant DNA**		

Zebrafish full-length BAX	This work	N/A
Zebrafish ΔBH3-BAX	This work	N/A
Zebrafish MOM-ΔBH3-BAX	This work	N/A
Zebrafish ER-ΔBH3-BAX	This work	N/A
Zebrafish MOM-full-length BAX	This work	N/A
Zebrafish TMBIM3	This work	N/A
Zebrafish TMBIM6	This work	N/A
Venus-V2A-BH4 (from BC-L2)	This work	N/A
pTol2-PhiC31LS-BH:GFP-mfap4:Mito-GCaMP3	This work	N/A
pME mitoGCaMP3	D. Raible Laboratory; Esterberg et al., 2014	N/A
pTol2 PhiC31LS BH NewMCS (cmlc2:eGFP)	This work	N/A
pTol2 PhiC31LS BH NewMCS (cmlc2:RFP)	This work	N/A
pTol2 mfap4:TdTomato-CAAX	D. Tobin Laboratory; Walton et al., 2015	N/A
pSB_PhiC31LandingSite	Kirchmaier et al., 2013	Addgene #48875
pCS2P+	Marc Kirschner	Addgene #17095

**Software and Algorithms**		

NIS-Elements	Nikon	N/A
Imaris	Bitplane	N/A
Prism	GraphPad	N/A
ImageJ	https://imagej.nih.gov/ij/	N/A
FPC (ImageJ); macro for quantification of bacterial burden by fluorescence imaging used in this work for quantification of camptothecin-induced apoptosis	Takaki et al., 2013	N/A
CorelDraw X5	Corel	N/A

### Lead Contact and Materials Availability

Further information and requests for resources and reagents should be directed to and will be fulfilled by the Lead Contact, Lalita Ramakrishnan (lr404@cam.ac.uk).

### Experimental Model and Subject Details

#### Zebrafish husbandry and infections

Zebrafish husbandry and experiments were conducted in compliance with guidelines from the UK Home Office (experiments conducted in the University of Cambridge) and with the U.S. Public Health Service Policy on Humane Care and Use of Laboratory Larvae using protocols approved by the Institutional Animal Care and Use Committee of the University of Washington (generation of *Baxa*^*rr1*^ and *Baxb*^*rr10*^ mutant lines at the University of Washington). Zebrafish AB wild-type strain (Zebrafish International Resource Center) and transgenic or mutant lines in the AB background were used, including *Tg(mpeg1:YFP)*^*w200*^ (with yellow fluorescent macrophages) (Roca and Ramakrishnan, 2013), *Tg(mpeg1:Brainbow)*^*w201*^ (with red fluorescent macrophages) (Pagán et al., 2015), *Tg(BH:GFP-mfap4:Mito-GCaMP3)* (expressing the genetically Ca^2+^ sensor targeted to the mitochondrion in macrophages) (this work), *baxa*^*rr1*^ (this work), *baxb*^*rr10*^ (this work) and *ripk3*^*rr7*^ (this work). All zebrafish lines were maintained in buffered reverse osmotic water systems and were exposed to a 14 hr light - 10 hr dark cycle to maintain proper circadian conditions. Fish were fed twice daily a combination of dry food and brine shrimp. Zebrafish embryos were housed in fish water (reverse osmosis water containing 0.18 g/l Instant Ocean) at 28.5°C. Embryos were maintained in 0.25 μg/ml methylene blue from collection to 1-day post-fertilization (dpf). 0.003% PTU (1-phenyl-2-thiourea, Sigma) was added from 24 h post-fertilization (hpf) on to prevent pigmentation. Larvae (of undetermined sex given the early developmental stages used) were anesthetized in fish water containing 0.025% tricaine (Sigma) and infected at 48 hpf via caudal vein (CV) injection using single-cell suspensions of Mm or Mtb of known titer (Takaki et al., 2012, Takaki et al., 2013). Larvae were randomly allotted to the different experimental conditions. Number of larvae to be used for each experiment was guided by pilot experiments.

Generation of the transgenic zebrafish line *Tg(BH:GFP-mfap4:Mito-GCaMP3)*: the plasmid pTol2-PhiC31LS-BH:GFP-mfap4:Mito-GCaMP3 was generated by PCR amplifying the mito-GCaMP3 cassette from the plasmid pME mitoGCaMP3 (Esterberg et al., 2014) and cloning into a Tol2 plasmid with a green bleeding heart cassette (cmlc2:eGFP) for screening [pTol2 PhiC31LS BH NewMCS (cmlc2:eGFP)] followed by cloning of the mfap4 promoter (Walton et al., 2015). The plasmid pTol2-PhiC31LS-BH NewMCS (cmlc2:RFP) was generated by NEBuilder HiFi DNA Assembly following the manufacturer protocol by combining the backbone of the Tol2 plasmid pTol2 mfap4:TdTomato-CAAX (Walton et al., 2015) with sequence containing the bleeding heart and Phi31 Landing cassettes from the plasmid pSB_PhiC31LandingSite (Kirchmaier et al., 2013) (Addgene plasmid 48875) where HA tag was previously removed and a new multi-cloning site inserted. A version of pTol2 PhiC31LS BH NewMCS with eGFP expression in the myocardium [pTol2 PhiC31LS BH NewMCS (cmlc2:eGFP)] was generated by replacing RFP with eGFP by NEBuilder HiFi DNA Assembly. The pTol2-PhiC31LS-BH:GFP-mfap4:Mito-GCaMP3 plasmid was injected along with transposase mRNA into one- to two-cell-stage embryos of the wild-type AB strain as previously described (Suster et al., 2011) using injection mix (1 × Tango Buffer (Thermo Scientific) containing 2% phenol red sodium salt solution (Sigma)). Putative founders were identified by GFP expression in the heart and crossed to wild-type AB zebrafish.

BAXA-, BAXB- and RIPK3-deficient zebrafish lines were generated by using the CRISPR/Cas9 system (Irion et al., 2014) at the University of Washington. The mutation consists of a single nucleotide (G) deletion in exon 3 of baxa, a deletion of three nucleotides (TGG) and insertion of thirteen nucleotides (AATAAAGAGGTGA) in exon 3 of baxb and a deletion of eleven nucleotides (TCTGCTGCAGA) and insertion of eighteen nucleotides (GAACGTTTTTAGAAAGCA) in exon 2 of RIPK3. All these mutations result in a frameshift and multiple premature STOP codons. *baxa*^*rr1*^, *baxb*^*rr10*^ and *ripk3*^*rr7*^ lines were genotyped by high-resolution melt analysis (HRMA) (Garritano et al., 2009) of PCR products (see Key Resources Table for sequences) on a CFX Connect thermocycler (BioRad).

#### THP-1 cell culture and differentiation into macrophages

THP-1 cells (ATCC TIB-202) were maintained in RPMI 1640 culture media supplemented with 10% fetal bovine serum (FBS, GIBCO) and 1% L-glutamine (Sigma). THP-1 cells were differentiated into macrophages by stimulation with 200 nM phorbol 12-myristate 13-acetate (PMA) (Sigma) in 24 well black optical bottom plates (Perkin Elmer), at a density of 3 × 10^5^ macrophages per well. On day 3 post-differentiation, the resulting adherent cells were washed with fresh medium and incubated for 3-5 days before infection (protocol modified from (Daigneault et al., 2010)).

### Method Details

#### Bacterial strains

WT *M. marinum* M strains (ATCC #BAA-535) expressing tdTomato, tdKatushka, or EBFP2 under the msp12 promoter (Takaki et al., 2013) were grown under hygromycin B (Formedium) selection in 7H9 Middlebrook medium (Difco) supplemented with oleic acid, albumin, dextrose, and Tween-80 (Sigma) (Takaki et al., 2013).

*M. tuberculosis ΔleuD ΔpanCD* double auxotroph expressing mCherry or GFP under control of the *msp12* promoter were grown under hygromycin B (Formedium) and kanamycin selection in Middlebrook 7H9 broth (Difco) supplemented with oleic acid, albumin, dextrose, catalase, Tween-80 (Sigma), 0.05 mg/ml L-leucine and 0.024 mg/ml calcium pantothenate (Sigma) (Sampson et al., 2011).

#### TNF and drug administration to zebrafish larvae

0.5 ng of recombinant zebrafish soluble TNF (Roca et al., 2008) or vehicle was microinjected into the caudal vein of each animal 1-day post infection to create TNF-high animals and matched controls (Tobin et al., 2012). To assess drug treatment in infected fish, equivalently infected sibling larvae were mixed in a Petri dish and held at 28.5°C until they were then randomly allocated to the drug-treated or control. DMSO (Sigma) was kept at 0.5% in all conditions when drugs were being used. All drugs were dissolved in DMSO (Sigma) or water and kept in small aliquots at −20°C. The rationale for the doses used in this work was based on previous studies or pilot experiments, using the minimum effective concentration. None of these concentrations showed toxic effects in the larvae at the end of the experiment. All drugs were administered by adding them to the fish water. BI-6C9 (5 μM) (Sigma), BCB (Bax Channel Blocker) (10 μM) (Alfa Aesar) and alisporivir (10 μM) (Novartis) were administered 4 h after infection and removed 24 h post-TNF administration. Pepstatin A (7.5 μM) (Sigma), E64d (1 μg/ml) (Sigma), Ru360 (2 μM) (VWR International), thapsigargin (1.5 μM) (Sigma), desipramine (10 μM) (Sigma), necrostatin-1 (10 μM) (Alfa Aesar), amifostine (40 μM) (Cambridge Bioscience), NAC (30 μM) (Cambridge Bioscience), mitotempo (20 μM) (Cambridge Bioscience), GSH (20 μM) (Cambridge Bioscience), xestospongin C (5 μM) (Cambridge Bioscience), ryanodine (2 μM) (Generon), dantrolene (5 μM) (Sigma), 4-Chloro-*m*-cresol (4CmC) (10 μM) (Sigma), nifedipine (dihydropyridine) (3 μM) (Cambridge Bioscience), diltiazem (benzothiazepine) (5 μM) (Cambridge Bioscience) and verapamil (phenylalkylamine) (10 μM) (Fischer Scientific) were administered 5 h prior and removed 24 h post-TNF administration. For experiments quantifying mitochondrial ROS production or mitochondrial Ca^2+^ overload, drugs were administered 4 h after infection or 5 h prior to TNF administration as detailed before and maintained during imaging.

#### Morpholino knockdown

Cathepsin D-splice-blocking and BID-, cyclophilin D- and PGAM5-translation-blocking morpholinos (see Key Resources Table for sequences) (Gene Tools) were diluted in injection solution and approximately 2-4 nL were injected into the yolk of one- to two-cell-stage embryos (Tobin et al., 2012). The concentration in the injection solution for each morpholino was 0.2 mM for Cathepsin D and 0.15 mM for BID, cyclophilin D and PGAM5.

#### Synthetic mRNA synthesis and microinjection

The sequences for the ORFs for acid ceramidase, BAXA, BCL-2 and TMBIM3 and 6 were obtained by PCR from zebrafish cDNA. The ORF for acid ceramidase was then cloned into the plasmid pCS2P+ and mRNA was generated with T7 (Roca and Ramakrishnan, 2013). The T7 promoter followed by the Kozac sequence 5′-GCCGCCACC-3′ was inserted before the start codon by PCR for all versions of BAXA, Venus-2A-BH4 (from BCL-2) and TMBIM3 and 6. ΔBH3-BAX was generated by PCR by removing the BH3 domain. MOM-BAX and MOM-ΔBH3-BAX were generated by PCR by removing the Stop codon of BAX and fusing a linker sequence G_4_SG_4_SG_4_ followed by the MOM-targeting sequence from the protein ActA from *Listeria monocytogenes* (Zhu et al., 1996) and a Stop codon using as a template full-length or ΔBH3-BAX, respectively. ER-ΔBH3-BAX was generated by PCR by removing the Stop codon of BAX and fusing the linker sequence G_4_SG_4_SG_4_ followed by the ER-targeting sequence from the rat protein cytochrome b5 (Zhu et al., 1996) and a Stop codon using as a template ΔBH3-BAX. Venus-2A-BH4 (from BCL-2) was generated by PCR. For larval survival experiments, the sequences for BAX and ΔBH3-BAX were cloned in the plasmid pCS2P+ downstream and in frame with the sequence for venus-V2A to ensure expression of the constructs. RNA was synthesized using the mMessage mMachine kit (Ambion) and the polyA Tailing kit (Ambion). 2-4 nL were injected into the yolk of one- to two-cell-stage embryos at different concentrations in injection solution.

#### Zebrafish larvae microscopy

Fluorescence microscopy was performed as described (Takaki et al., 2013). Quantification of camptothecin-induced apoptosis by acridine orange staining and assessments of mycobacterial cording were performed with a Nikon Eclipse Ti-E inverted microscope fitted with 4x and 10x objectives. For laser scanning confocal microscopy, anesthetized larvae were embedded in 1.5% low-melting-point agarose on optical bottom plates or dishes (MatTek Corporation). For long term microscopy, agarose was covered with fish water containing 0.007% Tricaine. A Nikon A1R confocal microscope with a 203 Plan Apo 0.75 NA objective was used to generate 35-40 μm z stacks consisting of 0.3-2 μm optical sections. The galvano scanner was used for all static imaging and for time-lapse imaging of the CHT. Time-lapse images were taken at different intervals for up to 5 hr. Data were acquired with NIS Elements (Nikon). A heating chamber (Okolab) adapted to the microscope was used to maintain temperature constantly at 28.5°C during the imaging process. In Figure 1B, a single z image is shown for the control panels to appreciate cellularity and maximum intensity projections for TNF-high panels to appreciate cording. Confocal images are pseudocolored to facilitate visualization.

#### Embryo survival assay

*In vitro*-transcribed mRNA’s for venus, venus-2A-baxa, venus-2A-ΔBH3-baxa and venus-2A-MOM-baxa were diluted in injecting solution and injected into the yolk of one- to two-cell-stage embryos (Tobin et al., 2012) at a concentration of 300ng/μl. Embryo survival was monitored 5 and 24 h post injection.

#### Assessment of camptothecin-induced apoptosis in whole zebrafish larvae

2 dpf larvae were treated by bath with 500 nM camptothecin (Alfa Aesar) or DMSO (0.5% final concentration) and incubated for 6 h at 28.5°C (Langheinrich et al., 2002). Then all larvae from each treatment were transferred to 1.5 mL centrifuge tubes containing 1 mL of fish water and 2.4 μg/ml acridine orange (Sigma) and incubated protected from the light with rotation at room temperature for 30 min (Paquet et al., 2009). Finally, larvae were washed twice in fish water with tricaine and acridine orange fluorescence was analyzed by microscopy as a readout for apoptosis. ImageJ was used to quantify mean gray value for each animal as indicated in Figure S1.

#### Mitochondrial Ca^2+^ overload detection and quantification assay in zebrafish larvae

Mitochondrial Ca^2+^ overload was assayed by the increase in fluorescence intensity of the cell permeant mitochondrion-targeted Ca^2+^ indicator Rhod-2 AM (red fluorescence) (Pozzan and Rudolf, 2009) (Fisher Scientific) or the genetically encoded Ca^2+^ reporter GCaMP3 (green fluorescence) targeted to the mitochondrion [*Tg(BH:GFP-mfap4:Mito-GCaMP3)*]. *Tg(mpeg1:YFP)*^*w200*^ or *Tg(mpeg1:Brainbow)*^*w201*^; *Tg(BH:GFP-mfap4:Mito-GCaMP3)* larvae were infected with 90-120 EBFP2-expressing Mm or 185-200 GFP-expressing Mtb. To calculate the percentage of macrophages positive for Rhod-2, 1 day post infection (dpi) *Tg(mpeg1:YFP)*^*w200*^ larvae were microinjected via CV with a solution containing TNF and 31.25 μg/ml Rhod-2 or a solution containing vehicle for TNF and Rhod-2 only. 50 μg Rhod-2 were diluted in 20 μL DMSO and stored in 1 μL aliquots at −20°C and protected from light. Then Rhod-2 was diluted 1:40 in PBS and added 1:2 to the TNF solution. To quantify increased mitochondrial Ca^2+^ concentration, 1 dpi *Tg(mpeg1:Brainbow)*^*w201*^; *Tg(BH:GFP-mfap4:Mito-GCaMP3)* larvae were microinjected via CV with TNF. After TNF or TNF and Rhod-2 administration, larvae were prepared for time-lapse confocal imaging. Time-lapse images were taken at different intervals for 5 hr. Mitochondrial GCaMP3 fluorescence was quantified as maximum fluorescence intensity per macrophage for a single time point using NIS-Elements. Mitochondrial Ca^2+^ overload quantified as total Rhod-2 positive macrophages per animal in the area indicated in Figure 3A was determined over a period of 5 h. When not otherwise stated in the figure legend, Rhod-2 positivity or mean of maximum mitoGCaMP3 fluorescence was quantified only in Mm- or Mtb-infected macrophages.

#### Protein sequence analysis

LALIGN (https://embnet.vital-it.ch/software/LALIGN_form.html) was used for global analysis without end-gap penalty of amino acid residue sequences. Percentage identity, percentage similarity and global/local score are shown in Figure S1A. Protein accession numbers: hBAX alpha (NM_138761.3), hBAX beta (NM_004324.3), hBAK1-202 (NM_001188.3), hBAK1-203 (ENST00000442998.6), zfBAXA (NM_131562), zfBAXB (NM_001013296).

#### Mitochondrial ROS quantification assay in zebrafish larvae

Mitochondrial ROS production was assayed by fluorescence intensity of two cell permeant fluorogenic probes for ROS which are targeted to the mitochondrion: MitoTracker Red CM-H_2_-Xros (red fluorescence) (Roca and Ramakrishnan, 2013) (Fisher Scientific) and MitoSOX (red fluorescence) (Esterberg et al., 2014) (Fisher Scientific). MitoTracker Red CM-H_2_-Xros fluoresces red upon oxidation by diverse ROS while MitoSOX requires oxidation selectively by superoxide, the proximal reactive oxygen species produced by mitochondrial electron transport complexes before becoming fluorescent after binding DNA (Murphy, 2009). *Tg(mpeg1:YFP)*^*w200*^ larvae were infected with 90-120 EBFP2-expressing WT Mm or 80 GFP-expressing Mtb. Larvae were microinjected 1 dpi via CV with a solution containing TNF and 50 μΜ MitoTracker Red CM-H_2_-Xros or 68 μΜ MitoSOX or a solution containing vehicle for TNF and MitoTracker Red CM-H_2_-Xros or MitoSOX only. After TNF administration, larvae were prepared for confocal imaging and maintained within a heated incubation chamber (Oko-labs) attached to the confocal. Time-lapse images were taken at different intervals for 2-3 hr starting 40–60 min after TNF administration. MitoTracker Red CM-H_2_-Xros or MitoSOX were diluted in DMSO and stored in 1 μL 5 mM and 2.5 mM aliquots, respectively, at −20°C and protected from light. Then both probes were further diluted in PBS and added to the TNF solution. Mitochondrial ROS production was quantified as MitoTracker Red CM-H_2_-Xros or MitoSOX maximum fluorescence intensity per macrophage using NIS-Elements. When not otherwise stated in the figure legend, mean of maximum MitoTracker Red CM-H_2_-Xros fluorescence was quantified only in Mm- or Mtb-infected macrophages. The use of the heating chamber allowed us to quantify mitochondrial ROS production under more physiological conditions: we had previously showed that ROS production occurred 3 h post TNF administration (Roca and Ramakrishnan, 2013). Those results were obtained without a heating chamber, thus keeping larvae at room temperature (around 20°C). By keeping the larvae at 28.5°C we could now show that mitochondrial ROS production occurs within an hour after TNF administration.

#### THP-1 macrophage infection, TNF and drug administration and quantitation of cell death

On day of infection, single cell suspensions of tdKatushka-expressing WT Mm or mCherry-expressing *M. tuberculosis ΔleuD ΔpanCD* double auxotroph were diluted in RPMI 1640 containing 10% FCS to a concentration of ∼6 × 10^5^ C.F.U. per mL, corresponding to an MOI of 1. Adherent differentiated THP-1-derived macrophages were incubated with mycobacteria-containing medium or fresh medium for 3-4 hr at 33°C (Mm) or 37°C (Mtb), then washed and incubated in fresh medium at 33°C (Mm) or 37°C (Mtb) overnight. The next day, human recombinant TNF (Sigma) in a solution of 5% trehalose/PBS (Sigma) was added to treatment wells for a final concentration of 10ng/ml and cells were then incubated for 5 h. In experiments with small molecule inhibitors 10 μM Necrostatin-1, 10 μM Nec-1 s (Cambridge Bioscience), 10 μM Q-VD-OPh (Source Bioscience), 10 μM Z-VAD-FMK (Cambridge Bioscience), 10 μM BI-6C9, 10 μM dantrolene and 2 μM Ru360), cells were pre-incubated with drug or 0.1% DMSO vehicle control for 1 hr prior to TNF addition. In experiments with LTCC inhibitors (10 μM diltiazem, 5 μM nifedipine, and 5 μM verapamil), drug or 0.1% DMSO vehicle control were added immediately after Mm infection and incubated overnight before TNF addition. To identify necrotic cell death, SYTOX® Green Nucleic Acid Stain (Life Technologies), a green-fluorescent stain that is impermeant to live cells, was added to culture medium at least 30 min prior to imaging. Macrophages were imaged using a Nikon Ti-E inverted microscope fitted with 20x objective and 2-5 arbitrary images per well acquired with NIS Elements (Nikon). Cell necrosis was quantified by counting SYTOX® Green fluorescence-positive infected and uninfected cells in each image using ImageJ or Imaris (Bitplane Scientific Software), and the total percentage per treatment well of SYTOX+ infected or SYTOX+ uninfected cells calculated by summing values obtained from all images per well. To calculate the ratios of Mm infected +TNF versus infected –TNF, infected versus uninfected dead within TNF-treated wells, and control +TNF versus control –TNF (Figure 7C), mean values were calculated for all conditions in each independent experiment (Table S2).

### Quantification and Statistical Analysis

The following statistical analyses were performed using Prism 5.01 (GraphPad): One sample t test, one-way ANOVA with Bonferroni’s or Tukey’s post-test, Fisher’s exact test and Student’s unpaired t test. Error bars represent standard error of mean. Post-test *p value*s are as follows: Not significant, ^∗^ p < 0.05; ^∗∗^ p < 0.01; ^∗∗∗^ p < 0.001; ^∗∗∗∗^ p < 0.0001. The statistical tests used for each figure can be found in the corresponding figure legend. Where the *n* value is given and not represented graphically in the figure, *n* represents the number of zebrafish used for each experimental group.

### Data and Code Availability

The following software was used: NIS-Elements (image acquisition in wide-field and confocal microscopy), Imaris (image analysis of macrophage death), Corel Draw (figure preparation) and ImageJ (image analysis of macrophage death and quantification of camptothecin-induced apoptosis in zebrafish larvae); see Key Resources Table for more information.

#### Materials and Data Availability

Materials and Data will be made available upon request.

## Supplemental Information

Document S1. Tables S1–S2

Table S3. Gene Symbols and Names Used for Zebrafish Mutant and Overexpression Lines, Related to STAR Methods
