# Supplementary material for: TNF Induces Pathogenic Programmed Macrophage Necrosis in Tuberculosis through a Mitochondrial-Lysosomal-Endoplasmic Reticulum Circuit
Source: Cell. 2019 Sep 5;178(6):1344–1361.e11. doi: 10.1016/j.cell.2019.08.004 (PMC6736209; doi:10.1016/j.cell.2019.08.004)
Supplement: Document S1. Tables S1–S2 [file mmc1.pdf]

Cell, Volume *178*

Supplemental Information

TNF Induces Pathogenic Programmed Macrophage Necrosis in Tuberculosis through a Mitochondrial-Lysosomal-Endoplasmic Reticulum Circuit

Francisco J. Roca, Laura J. Whitworth, Sarah Redmond, Ana A. Jones, and Lalita Ramakrishnan

SUPPLEMENTAL DATA TITLES AND LEGENDS

Table S1 (related to Figure 4 and Figure 7). Expression of ryanodine receptor isoforms in human macrophages and zebrafish blood monocytes.

<table>
<tr><th colspan="2">HUMAN</th><th colspan="2">ZEBRAFISH</th></tr>
<tr><td>Homolog</td><td>Expression</td><td>Homolog</td><td>Expression in blood mfap4+ cells (Athanasiadis et al., 2017)</td></tr>
<tr><td rowspan="2">RyR1</td><td rowspan="2">Macrophages:
(EMBL-EBI expression atlas)
THP1:
Protein Atlas
(https://www.proteinatlas.org)</td><td>RyR1a</td><td>Yes (high)</td></tr>
<tr><td>RyR1b</td><td>nd</td></tr>
<tr><td rowspan="2">RyR2</td><td rowspan="2">Lung macrophages
(www.proteinatlas.org)</td><td>RyR2a</td><td>nd</td></tr>
<tr><td>RyR2b</td><td>Yes (high)</td></tr>
<tr><td>RyR3</td><td>No data available</td><td>RyR3</td><td>Yes (low)</td></tr>
</table>

Table S2 (related to Figure 7). THP-1 macrophage death (%) in all experiments.

Experiment Code	UNINFECTED WELL, NO TNF	UNINFECTED WELL, +TNF	UNINFECTED CELLS, NO TNF	INFECTED CELLS, NO TNF	UNINFECTED CELLS, +TNF	INFECTED CELLS, +TNF	INF+TNF/INF-TNF	INF+TNF/Uninf+TNF within well ratio	UNINF+TNF/UNINF-TNF (ALL UNINFECTED WELLS)	INF-TNF/UNINF-TNF within well ratio	UNINF+TNF/UNINF-TNF	J / L	I/M
UWS003	0.70	1.40	16.00	11.10	14.00	47.60		3.40		0.69			
	0.60	0.60	12.70	4.70	15.60	30.70		1.97		0.37			
mean	*0.65*	*1.00*	*14.35*	*7.90*	*14.80*	*39.15*	*4.96*	***2.68***	*1.54*	*0.53*	*1.03*	*5.05*	*4.81*
UWS004	7.71	-	12.90	13.73	13.04	24.60		1.89		1.06			
	6.46	-	9.68	11.85	4.00	22.54		5.64		1.22			
mean	*7.09*	-	*11.29*	*12.79*	*8.52*	*23.57*	*1.84*	***3.76***	*n/a*	*1.14*	*0.75*	*3.29*	*2.44*
CMB008	14.25	12.00	9.26	13.53	27.76	35.67		1.28		1.46			
	-	-	-	-	12.09	31.29		2.59					
mean	-	-	-	-	*19.93*	*33.48*	*2.47*	***1.94***	*0.84*		*2.15*	*1.33*	*1.15*
CMB010	6.96	7.03	11.11	16.67	11.20	46.81		4.18		1.50			
	11.62	12.23	9.20	18.46	14.34	39.33		2.74		2.01			
	7.00	16.14	17.48	18.42	27.96	57.53		2.06		1.05			
mean	*8.53*	*11.80*	*12.60*	*17.85*	*17.83*	*47.89*	*2.68*	***2.99***	*1.38*	*1.52*	*1.42*	*1.97*	*1.90*
CMB018	5.90	11.31	7.97	7.88	30.47	48.84		1.60		0.99			
	9.67	9.61	9.41	8.64	11.23	27.91		2.49		0.92			
mean	*7.79*	*10.46*	*8.69*	*8.26*	*20.85*	*38.38*	*4.65*	***2.04***	*1.34*	*0.95*	*2.40*	*2.14*	*1.94*
CMB019	6.58	7.46	8.04	5.10	13.27	34.31		2.59		0.63			
	8.41	19.75	13.81	16.20	15.70	30.41		1.94		1.17			
mean	*7.50*	*13.61*	*10.93*	*10.65*	*14.49*	*32.36*	*3.04*	***2.26***	*1.82*	*0.90*	*1.33*	*2.50*	*2.29*
CMB021	13.16	8.13	9.33	3.73	14.38	10.81		0.75		0.40			
	11.05	8.98	20.73	5.26	20.78	13.98		0.67		0.25			
	7.82	13.15	13.38	7.69	24.62	13.43		0.55		0.57			
mean	*10.68*	*10.09*	*14.48*	*5.56*	*19.93*	*12.74*	*2.29*	***0.66***	*0.94*	*0.41*	*1.38*	*1.60*	*1.67*
CMB029	25.58	28.79	19.80	25.00	30.25	32.26		1.07		1.26			
	16.46	25.93	19.93	33.60	22.90	30.44		1.33		1.69			
mean	*21.02*	*27.36*	*19.86*	*29.30*	*26.57*	*31.35*	*1.07*	***1.20***	*1.30*	*1.47*	*1.34*	*0.81*	*0.80*

Experiment Code	UNINFECTED WELL, NO TNF	UNINFECTED WELL, +TNF	UNINFECTED CELLS, NO TNF	INFECTED CELLS, NO TNF	UNINFECTED CELLS, +TNF	INFECTED CELLS, +TNF	INF+TNF/INF-TNF	INF+TNF/Uninf+TNF within well ratio	UNINF+TNF/UNINF-TNF (ALL UNINFECTED WELLS)	INF-TNF/UNINF-TNF within well ratio	UNINF+TNF/UNINF-TNF	J / L	I/M
CMB031	2.42	13.54	6.59	15.17	8.64	20.78		2.41		2.30			
	4.22	7.20	-	-	13.13	23.34		1.78					
mean	*3.32*	*10.37*	-	-	*10.89*	*22.06*	*1.45*	***2.09***	*3.12*		*1.65*	*0.91*	*0.88*
CMB040	2.49	3.44	4.17	7.69	5.42	5.95		1.10		1.84			
	7.62	2.09	2.94	11.54	10.81	15.46		1.43		3.93			
mean	*5.06*	*2.77*	*3.56*	*9.62*	*8.12*	*10.71*	*1.11*	***1.26***	*0.55*	*2.88*	*2.28*	*0.44*	*0.49*
CMB075	11.44	10.61	13.15	24.27	15.71	49.40		3.14		1.85			
	11.49	9.26	12.86	34.88	7.36	25.71		3.49		2.71			
	10.48	18.13	9.21	15.69	13.91	32.50		2.34		1.70			
mean	*11.14*	*12.67*	*11.74*	*24.95*	*12.33*	*35.87*	*1.44*	***2.99***	*1.14*	*2.09*	*1.05*	*1.43*	*1.37*
CMB076	11.89	7.25	10.20	22.90	3.40	14.90		4.38		2.25			
	11.00	8.05	11.60	16.10	7.90	31.40		3.97		1.39			
	12.43	14.12	17.16	28.40	12.10	43.10		3.56		1.66			
mean	*11.77*	*9.81*	*12.99*	*22.47*	*7.80*	*29.80*	*1.33*	***3.97***	*0.83*	*1.76*	*0.60*	*2.25*	*2.21*
CMB077	5.13	4.56	4.79	7.09	5.08	13.60		2.68		1.48			
(Mtb infection)	4.38	5.28	5.36	8.47	6.47	18.90		2.92		1.58			
	2.65	7.81	3.94	6.03	3.70	12.90		3.49		1.53			
	5.01	6.74	3.08	5.68	5.01	9.59		1.91		1.84			
	4.25	6.62	5.05	2.99	7.66	17.82		2.33		0.59			
	5.59	6.54	4.23	5.30	5.12	9.00		1.76		1.25			
mean	*4.50*	*6.26*	*4.41*	*5.93*	*5.51*	*13.64*	*2.30*	***2.51***	*1.39*	*1.38*	*1.25*	*1.82*	*1.84*
